# Ataxia-telangiectasia mutated (*ATM*) silencing promotes neuroblastoma progression through a *MYCN* independent mechanism

**DOI:** 10.18632/oncotarget.4061

**Published:** 2015-05-26

**Authors:** Stefano J. Mandriota, Linda J. Valentijn, Laurence Lesne, David R. Betts, Denis Marino, Mary Boudal-Khoshbeen, Wendy B. London, Anne-Laure Rougemont, Edward F. Attiyeh, John M. Maris, Michael D. Hogarty, Jan Koster, Jan J. Molenaar, Rogier Versteeg, Marc Ansari, Fabienne Gumy-Pause

**Affiliations:** ^1^ Department of Pediatrics, CANSEARCH Research Laboratory, Faculty of Medicine, University of Geneva, Geneva, Switzerland; ^2^ Department of Oncogenomics, Academic Medical Center, University of Amsterdam, The Netherlands; ^3^ Department of Clinical Genetics, Our Lady's Children's Hospital, Dublin, Ireland; ^4^ Division of Pediatric Hematology/Oncology, Harvard Medical School, Dana-Farber/Children's Hospital Cancer and Blood Disorders Center, Boston, MA, USA; ^5^ Department of Pathology, University Hospital of Geneva, Geneva, Switzerland; ^6^ Department of Pediatrics, Children's Hospital of Philadelphia and the University of Pennsylvania, Philadelphia, PA, USA; ^7^ Department of Pediatrics, Onco-hematology Unit, University Hospital of Geneva, Geneva, Switzerland

**Keywords:** ataxia-telangiectasia mutated, neuroblastoma, MYCN, 11q

## Abstract

Neuroblastoma, a childhood cancer with highly heterogeneous biology and clinical behavior, is characterized by genomic aberrations including amplification of *MYCN*. Hemizygous deletion of chromosome 11q is a well-established, independent marker of poor prognosis. While 11q22-q23 is the most frequently deleted region, the neuroblastoma tumor suppressor in this region remains to be identified. Chromosome bands 11q22-q23 contain *ATM*, a cell cycle checkpoint kinase and tumor suppressor playing a pivotal role in the DNA damage response. Here, we report that haploinsufficiency of *ATM* in neuroblastoma correlates with lower *ATM* expression, event-free survival, and overall survival. *ATM* loss occurs in high stage neuroblastoma without *MYCN* amplification. In SK-N-SH, CLB-Ga and GI-ME-N human neuroblastoma cells, stable *ATM* silencing promotes neuroblastoma progression in soft agar assays, and in subcutaneous xenografts in nude mice. This effect is dependent on the extent of *ATM* silencing and does not appear to involve *MYCN*. Our findings identify *ATM* as a potential haploinsufficient neuroblastoma tumor suppressor, whose inactivation mirrors the increased aggressiveness associated with 11q deletion in neuroblastoma.

## INTRODUCTION

Neuroblastoma (NB) is the most frequent solid tumor of infancy. It originates from the sympathetic nervous system (most frequently in adrenal glands), accounts for 8–10% of malignancies in childhood, and causes 15% of cancer-related deaths in children. Prognosis in NB is highly variable, ranging from spontaneous regression to highly aggressive disease, often resistant to multimodal therapy. From a cytogenetic point of view, NB characterized by the loss or gain of entire chromosomes often present with favorable prognostic features. In contrast, unbalanced gain or loss or chromosomal regions (in particular, the loss of chromosome 1p or 11q, the gain of 17q, or the amplification of the *MYCN* oncogene) have been associated with an adverse prognosis. Poor prognosis NB is subdivided into two main groups: NB with amplification of the *MYCN* oncogene (20% of cases) and NB with unbalanced loss of chromosome 11q (30–40% of cases). *MYCN* amplification and 11q loss occur together in NB very rarely (1.7% of cases, as opposed to an expected frequency of 8% if these two events occurred independently) suggesting that these two cytogenetic anomalies might be incompatible, for reasons that are currently unknown [1, 2].

The *MYCN* gene encodes N-myc, a helix-loop-helix/leucine zipper transcription factor frequently dysregulated in cancer, that controls the expression of several genes involved in cell cycle progression, cellular invasion, metabolism, and apoptosis. The observation that overexpression of *MYCN* or of its upstream positive regulator *LIN28B* targeted to the sympathetic adrenergic lineage of transgenic mice leads to the development of tumors closely resembling human NB [3, 4] supports the hypothesis that *MYCN* amplification causes NB in humans. Whereas *MYCN* amplification is a powerful prognostic marker in NB, a typical *MYCN* gene signature is found in both *MYCN* amplified NB and in a subset of *MYCN* non-amplified NB having post-transcriptionally stabilized N-myc protein or amplified *MYC*. This signature was more powerful, as a prognostic marker, than *MYCN* amplification [5].

Chromosome bands 11q22-q23, the region most frequently lost in NB, contain *ATM*, the gene mutated in ataxia telangiectasia (AT), an autosomal recessive syndrome characterized by neurodegeneration, oculocutaneous telangiectasia, radiosensitivity, immune deficiency, sterility, strong predisposition to lymphoid cancers and, at the cellular level, cell-cycle checkpoint defects and chromosomal instability. *ATM* encodes a homonymous Ser/Thr protein kinase that regulates cell cycle checkpoints, DNA repair, and apoptosis in response to DNA double-strand breaks (DSBs) by phosphorylating several hundred-protein substrates including p53 [6, 7]. Among the DSBs ATM responds to are those caused by activated cellular oncogenes, probably through the induction of proliferation stress. Once activated, the ATM pathway leads to cell cycle arrest, apoptosis or cellular senescence, the latter being a condition of permanent cell growth arrest in otherwise metabolically active cells [8]. Interestingly, N-myc downregulates ATM through the induction of miR-421 [9], suggesting that ATM downregulation is part of the *MYCN* dictated cellular transformation program in NB.

In addition to its prognostic value, 11q deletion might contribute to NB progression through the loss of 11q tumor suppressor(s). To investigate the possibility that alterations in *ATM* play a role in NB, we analyzed *ATM* gene status and expression in two panels of NB samples and in NB cell lines. Based on the results obtained, that demonstrated an association between *ATM* deletion, decreased *ATM* expression and poor prognosis, we mimicked the observed reduction in *ATM* expression in three human NB cell lines by stable *ATM* silencing.

## RESULTS

### *ATM* deletion correlates with lower *ATM* expression, event-free survival (EFS), and overall survival (OS)

**Figure 1:**
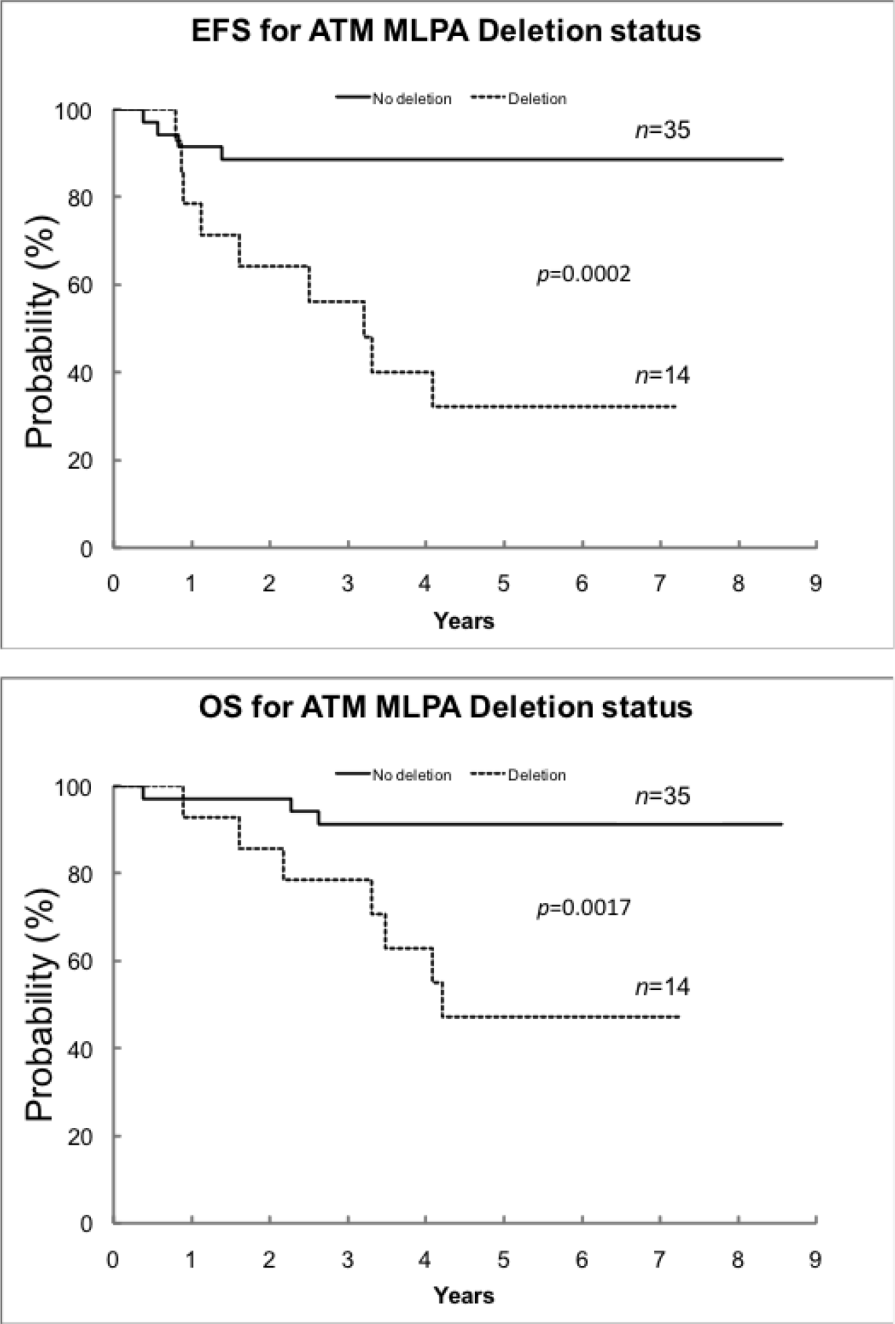
EFS and OS by *ATM* MLPA deletion status in the NB50 tumor set One patient was excluded because of *ATM* duplication. Deletion of *ATM* is significantly associated with lower event-free survival (EFS) and overall survival (OS) by using the Kaplan-Meier methods. Curves were compared using a log-rank test. EFS and OS are expressed as the estimate +/− the standard error. For EFS, the 5-year survival rate for no deletion was 89% ± 7% compared to 32% ± 15% for *ATM* deletion (*p* = 0.0002). For OS, the 5-year survival rate for no deletion was 91%± 6% compared to 47% ± 15% for *ATM* deletion (*p* = 0.0017).

**Figure 2:**
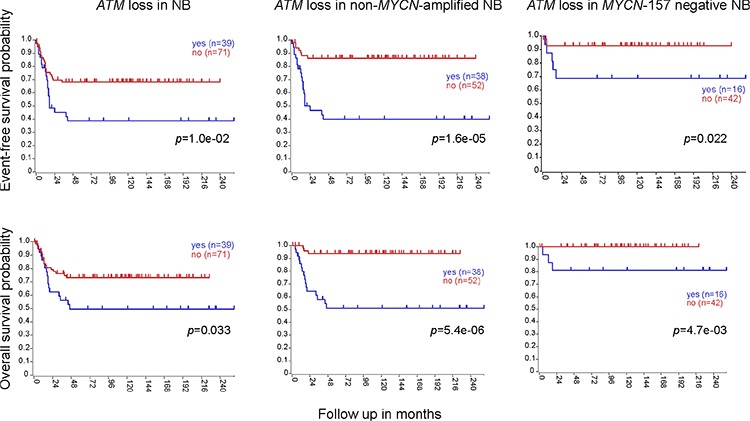
*ATM* deletion correlates with lower EFS and OS, independently of *MYCN* amplification Event-free survival (EFS) (upper panels) or overall survival (OS) (lower panels) analysis of *ATM* loss in the whole NB110 tumor set (left), its non-*MYCN*-amplified subset (center) or its *MYCN*-157-negative subset after K-means clustering with the *MYCN*-157 signature (right). *p* values were from log-rank test.

**Figure 3:**
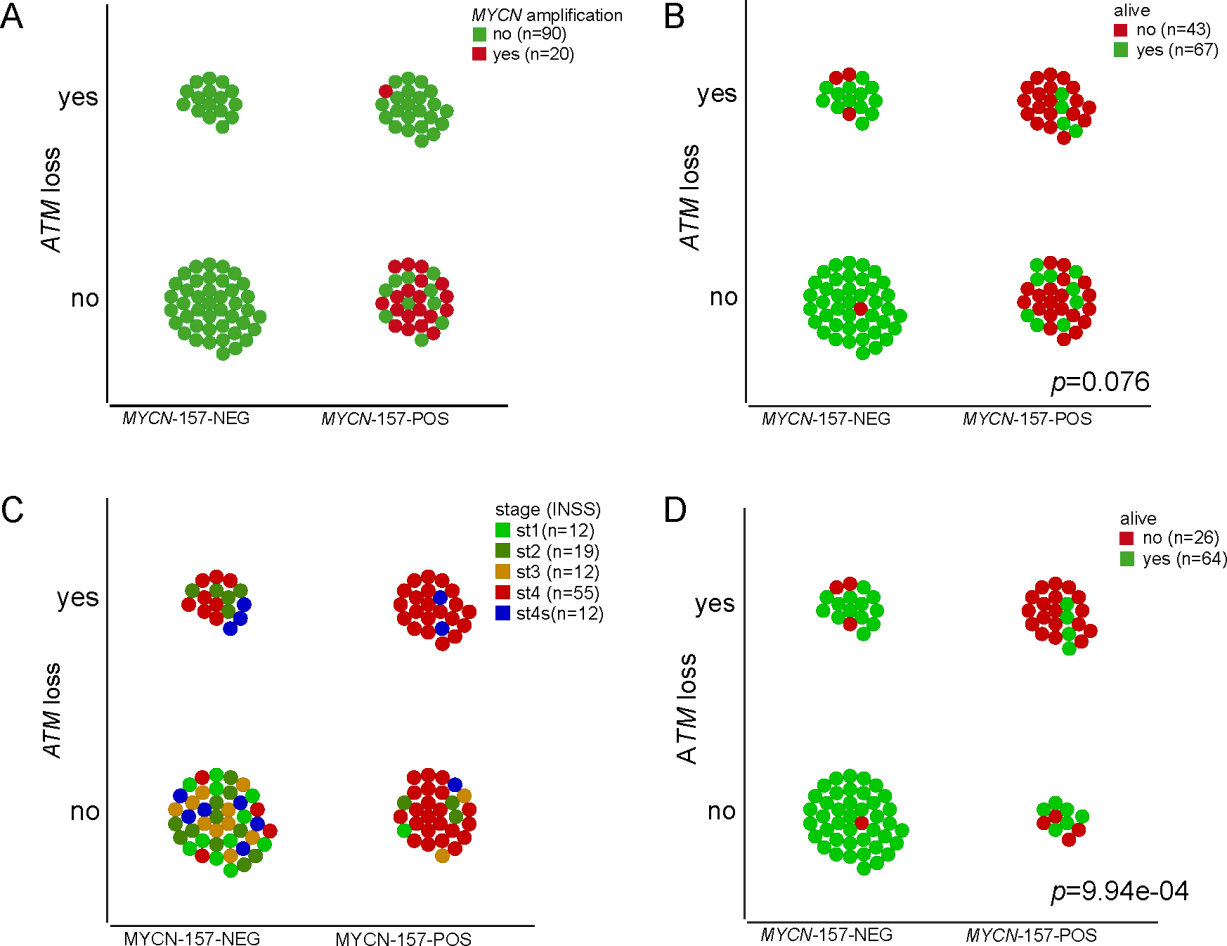
*ATM* deletion correlates with lower EFS and OS and with advanced tumor staging, independently of *MYCN* amplification The plots show 11q loss at the *ATM* locus versus K-means clustering of the NB110 tumor set using the *MYCN*-157-signature. Tumors were colored for *MYCN* amplification **(A)** survival **(B)** or INSS stage **(C). D.** Plot showing only non-*MYCN*-amplified samples colored for survival. *p* values are from Fisher's exact test.

**Figure 4:**
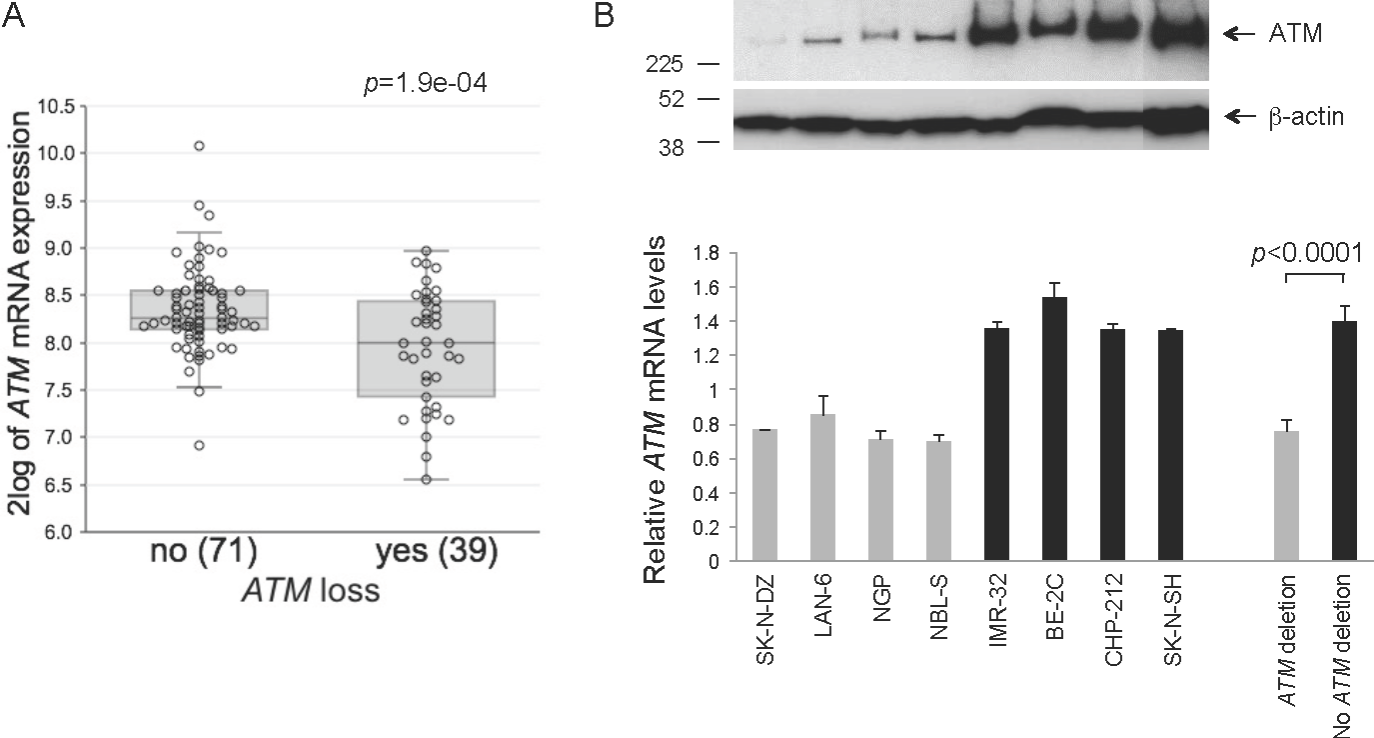
*ATM* expression levels in NB and NB cell lines **A.** *ATM* expression detected by Affymetrix profiling in 110 NB tumors separated in 2 groups by absence (no) or presence (yes) of loss of chromosome 11q at the *ATM* locus as determined by CGH analysis. Anova test. **B.** *ATM* mRNA (lower panel) levels were measured by real-time quantitative PCR in 50% confluent cultures of the indicated NB cell lines having (grey columns) or not (black columns) *ATM* deletion as assessed by FISH using a human *ATM* probe. The columns represent: (left) the normalized value ± SEM from three independent experiments for the indicated individual cell lines; (right) the grouped values for all the NB cell lines shown on the left having (grey column) or not (black column) *ATM* deletion, ± SEM. Two-tailed *t*-test. : parallel cultures were analyzed for ATM or β-actin expression by Western Blotting. Numbers on the left indicate kDa.

By full exome mutation screening using DHPLC, with the exception of a c.8147T > C (p.Val2716Ala) change, a missense mutation known to be pathogenic [10] in IMR-32 cells, we found no previously identified *ATM* mutations or gene hypermethylation in a panel of 16 NB cell lines (CHLA-171, IMR-32, LAN-1, NB16, NBL-S, NGP, SK-N-AS, SK-N-DZ, BE-2C, CHLA-79, CHP-212, CHP-901, KCNR, LAN-6, SK-N-FI, SK-N-SH), but several *ATM* rare variants (having minor allele frequency (MAF) < 0.01) of unknown significance (data not shown). No known *ATM* mutations, intragenic deletions/duplications or gene hypermethylation were found in a panel of 50 NB specimens (Supplementary Figure S1). The lack of known *ATM* mutations in this NB series is consistent with previous data [11–13]. The frequency and kind of *ATM* rare variants detected in NB specimens was similar to that found in a series of 60 healthy controls (data not shown), but 14/50 of the tumor samples or 6/16 of the cell lines considered (NB16, NBL-S, NGP, SK-N-AS, SK-N-DZ, LAN-6) were found to have a complete hemizygous *ATM* deletion as assessed by multiplex ligation-dependent probe amplification assay (MLPA). *ATM* deletion in the six NB cell lines was confirmed by *ATM* FISH (data not shown). Only one tumor had both *MYCN* amplification and *ATM* deletion (Supplementary Figure S1). *ATM* deletion was associated with lower EFS and OS (Figure 1). INSS stage (1, 2, 3 vs 4 and 1, 2 vs 3, 4) is statistically significantly associated with *ATM* deletion status, whereby *ATM* deletion is associated with higher stage [stages 1, 2, 3 versus 4 (*p* = 0.0099); stages 1, 2 versus 3, 4 (*p* = 0.0242)]. In a second series of NB (reported in ref. [5], plus additional samples) consisting of 110 specimens for which both *ATM* expression and *ATM* locus status were available, 11q deletion at the *ATM* locus correlates with lower EFS and OS (Figure 2, left), and this is independent of *MYCN* (Supplementary Figure S2). Of the 71 NB retaining *ATM* in this series, 3 had 11q loss outside the *ATM* locus. In general, tumors with a positive *MYCN*-157 gene signature [5] have either *MYCN* amplification or *ATM* loss and this correlates with OS and stage (Figure 3A–3C). When excluding *MYCN* amplified tumors, the prognostic value of *ATM* loss strongly increases (Figure 3D and Figure 2, centre) and this is independent of stage and *MYCN-157* signature (Supplementary Figure S2). Moreover, *ATM* loss also correlates with lower EFS and OS in tumors with a negative *MYCN-157* signature (Figure 2 right). In the NB110 tumor set, *ATM* mRNA levels were significantly reduced in the specimens carrying loss of *ATM* (Figure 4A). In cultured NB cell lines, *ATM* mRNA and protein levels were reduced in the cell lines carrying *ATM* deletion as assessed by *ATM* FISH and MLPA (Figure 4B). Altogether, these results raise the possibility that partial *ATM* loss might contribute to NB progression and suggest that, if this hypothesis is true, haploinsufficiency, with the consequent reduction in *ATM* expression, could be the mechanism involved.

### Stable *ATM* silencing promotes NB progression *in vitro* and *in vivo*

To investigate the possibility that decreased *ATM* expression contributes to NB progression, we generated NB cells stably expressing short hairpin (sh) RNAs against *ATM*. Using this approach, we previously reported the cell-type specific transforming effect of *ATM* inactivation in human mammary epithelial cells, which mirrors the breast cancer predisposition of AT carriers [14]. For the NB experiments, we selected the SK-N-SH cell line, characterized by the lack of *MYCN* amplification, diploid *ATM* gene status and functional ATM and p53 (ref. [15–17]; and Figure 4B).

**Figure 5:**
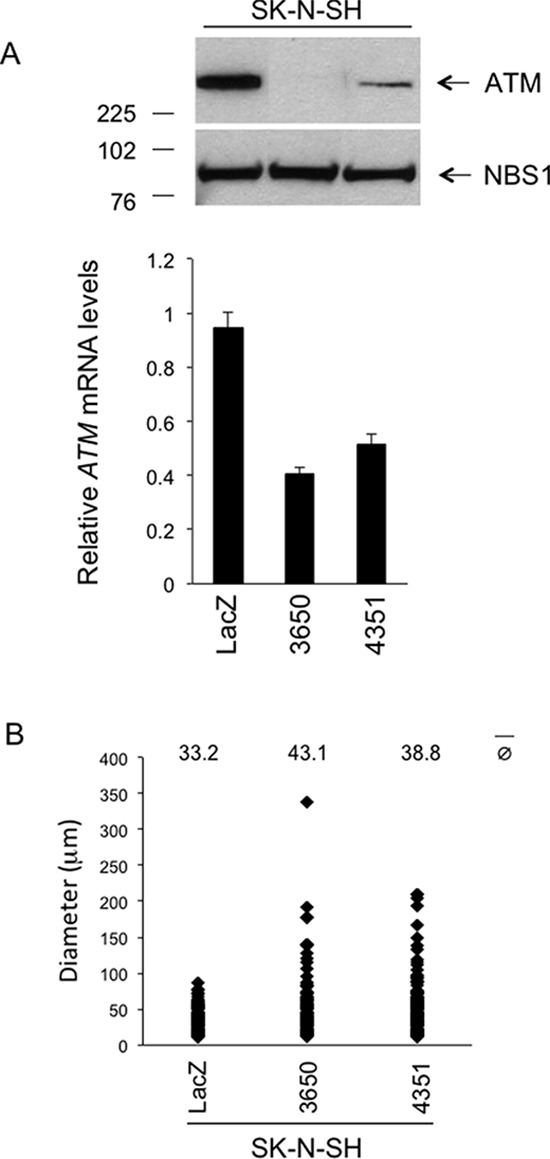
Phenotypic consequences of stable *ATM* silencing in the SK-N-SH cell line *in vitro* **A.** Western Blotting for ATM in SK-N-SH cells stably transfected with *ATM* shRNA vectors 3650, 4351 or with a LacZ shRNA vector as a control. NBS1 was used as a loading control. The graph (lower panel) shows the relative *ATM* mRNA levels as assessed by real-time quantitative PCR, ± SD from two different experiments. *p* 3650 *vs* LacZ < 0.001; *p* 4351 *vs* LacZ < 0.001 (two-tailed *t*-test). **B.** SK-N-SH cells stably transfected with *ATM* shRNA vectors 3650 or 4351, or with LacZ shRNA vector as a control, were resuspended in agarose gels at the density of 2 × 10^4^ cells/ml and grown for 14 days. The growth in agarose gels was quantified by measuring the diameter of the structures (single cells or multicellular colonies) formed after 14 days. At least 175 randomly selected structures per condition (single cells or multicellular colonies) from two independent experiments/condition were measured. *p* 3650 *vs* LacZ < 0.01; *p* 4351 *vs* LacZ < 0.05 (two-tailed *t*-test).

When compared to controls expressing LacZ shRNA (referred to as SK-N-SH^LacZ^), SK-N-SH cells stably expressing *ATM* shRNA 3650 (referred to as SK-N-SH^kd-3650^) have almost undetectable ATM protein, whereas SK-N-SH cells stably expressing *ATM* shRNA 4351 (referred to as SK-N-SH^kd-4351^) have an intermediate level of *ATM* silencing (Figure 5A, upper panel). *ATM* mRNA levels were correspondingly reduced in SK-N-SH^kd-3650^ and SK-N-SH^kd-4351^ cells compared to SK-N-SH^LacZ^ cells (Figure 5A, bottom panel).

**Figure 6:**
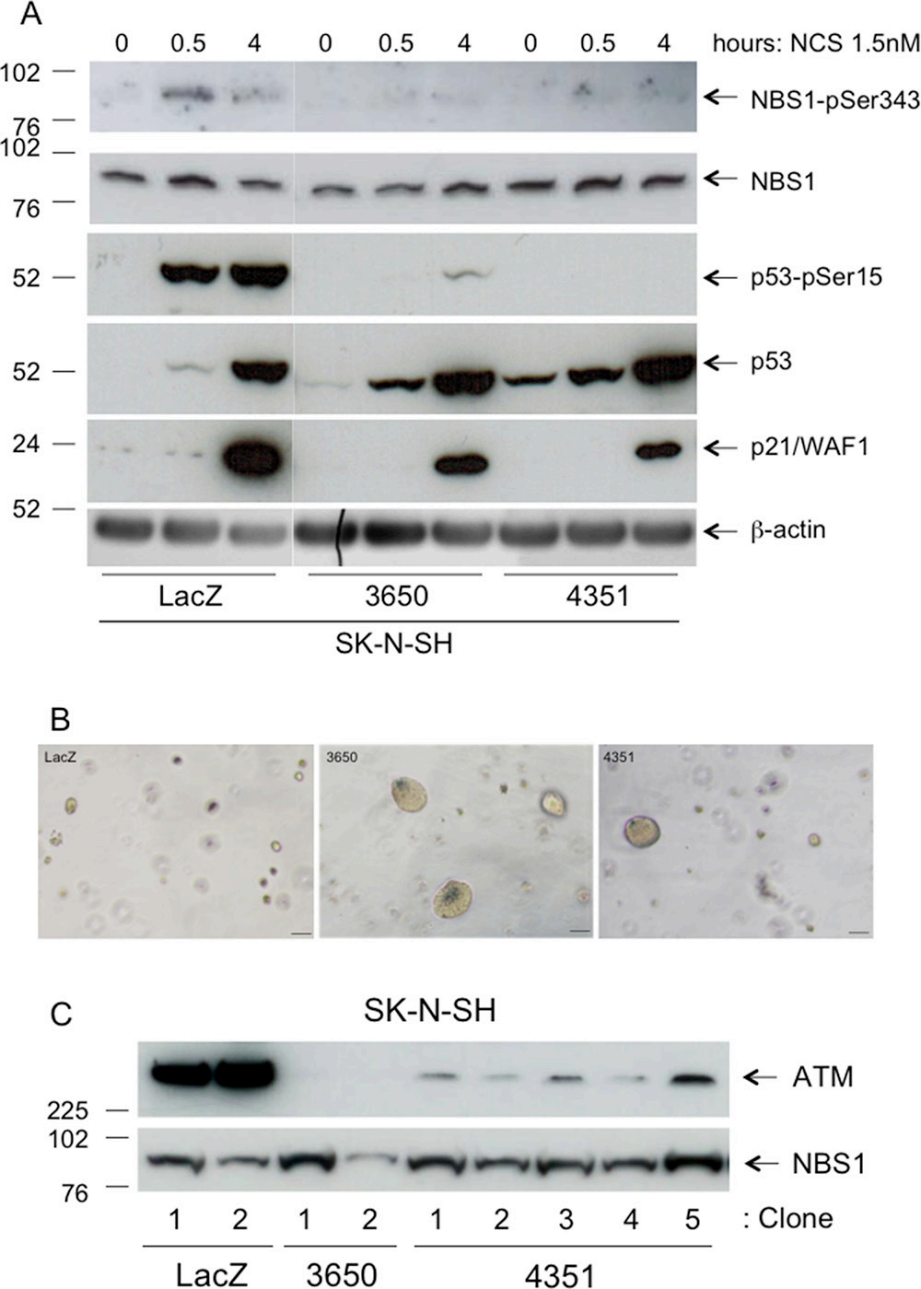
Characterization of SK-N-SH stable transfectants **A.** SK-N-SH cells stably transfected with *ATM* shRNA vectors 3650, 4351 or with a LacZ shRNA vector as a control were incubated in the presence of NCS 1.5nM for the indicated time points and analyzed for NBS1-pSer343, NBS1, p53-pSer15, p53, p21/WAF1 or β-actin levels by Western Blotting. **B.** SK-N-SH cells stably transfected with *ATM* shRNA vectors 3650 or 4351, or with LacZ shRNA vector as a control, were resuspended in agarose gels at the density of 2 × 10^4^ cells/ml and photographed under phase contrast after 14 days. Bar = 100 μm. **C.** Individual colonies of SK-N-SH cells (approximately 200 μm in diameter) stably transfected with *ATM* shRNA vectors 3650, 4351 or with LacZ shRNA vector were picked from the soft agar assay, put back in culture and analyzed by Western Blotting for ATM or NBS1 as soon as they reached confluence in 35 mm Petri dishes.

In response to DSBs ATM phosphorylates several protein substrates. This is followed by a transcriptional response mediated in part by p53, one of the ATM phosphorylation substrates and transcriptional effectors [18]. To assess whether reduced ATM expression consistently results in defective ATM substrate phosphorylation in response to DSB inducers, we analyzed the phosphorylation of two well-known ATM substrates – NBS1-Ser343 and p53-Ser15 – in SK-N-SH^kd-3650^, SK-N-SH^kd-4351^ or SK-N-SH^LacZ^ cells treated with neocarzinostatin (NCS), a chromoprotein enediyne antibiotic that specifically induces DSBs [19]. NCS increases NBS1-Ser343 or p53-Ser15 phosphorylation after 30min in SK-N-SH^LacZ^ cells. In contrast, phosphorylation of these substrates in NCS treated SK-N-SH^kd-3650^ or SK-N-SH^kd-4351^ cells was largely defective (Figure 6A). *CDKN1A* is a well-characterized p53 target gene encoding p21/WAF1, a cyclin-dependent kinase inhibitor that mediates p53-induced growth arrest in response to DNA damage [20]. Basal levels of p21/WAF1 were barely detectable in SK-N-SH^LacZ^ cells and undetectable in SK-N-SH^kd-3650^ and SK-N-SH^kd-4351^ cells (Figure 6A). Four hours after NCS treatment, p21/WAF1 was strongly induced in SK-N-SH^LacZ^ cells and to a lesser extent in SK-N-SH^kd-3650^ and SK-N-SH^kd-4351^ cells, consistent with defective phosphorylation of p53 (Figure 6A). Thus, as expected on the basis of their reduced level of ATM expression, SK-N-SH^kd-3650^ cells and SK-N-SH^kd-4351^ cells exhibit defective phosphorylation of NBS1–Ser343 and p53-Ser15 as well as reduced induction of p21/WAF1 in response to NCS.

To investigate the possibility that reduced expression of *ATM* confers a growth advantage to SK-N-SH cells *in vitro*, we compared SK-N-SH^LacZ^, SK-N-SH^kd-3650^ and SK-N-SH^kd-4351^ cells for their capacity to proliferate in the soft agar assay, a well-established method to measure cellular transformation *in vitro*. In these experiments, SK-N-SH^kd-3650^ cells and SK-N-SH^kd-4351^ cells form colonies larger than those formed by SK-N-SH^LacZ^ cells, the effect being more pronounced in SK-N-SH^kd-3650^ cells, which have the strongest silencing of *ATM* (Figure 5A, 5B; 6B). Since, as seen in SK-N-SH^kd-3650^ cells, almost complete silencing of *ATM* strongly promotes growth in agar (Figure 5B), and since SK-N-SH^kd-4351^ cells have partial *ATM* silencing (Figure 5A), one might argue that colonies formed in agar by SK-N-SH^4351^ cells originate from a particular cell subpopulation having a level of *ATM* expression lower than the average, detected by Western Blotting in the whole cell population (Figure 5A). To clarify this point, we picked individual colonies from the soft agar assay, put them back in culture and analyzed them by Western Blotting as soon as they reached confluence. When compared to cultures derived from SK-N-SH^LacZ^ cell colonies, all the cultures derived from SK-N-SH^kd-4351^ cell colonies analyzed retained a detectable level of ATM expression, like the original total cell population (Figure 6C, Figure 5A). In contrast, ATM protein remained undetectable in cultures derived from SK-N-SH^kd-3650^ cell colonies (Figure 6C, Figure 5A). Thus, partial silencing of *ATM* is sufficient to confer a proliferative advantage to SK-N-SH cells in the soft agar assay.

**Figure 7:**
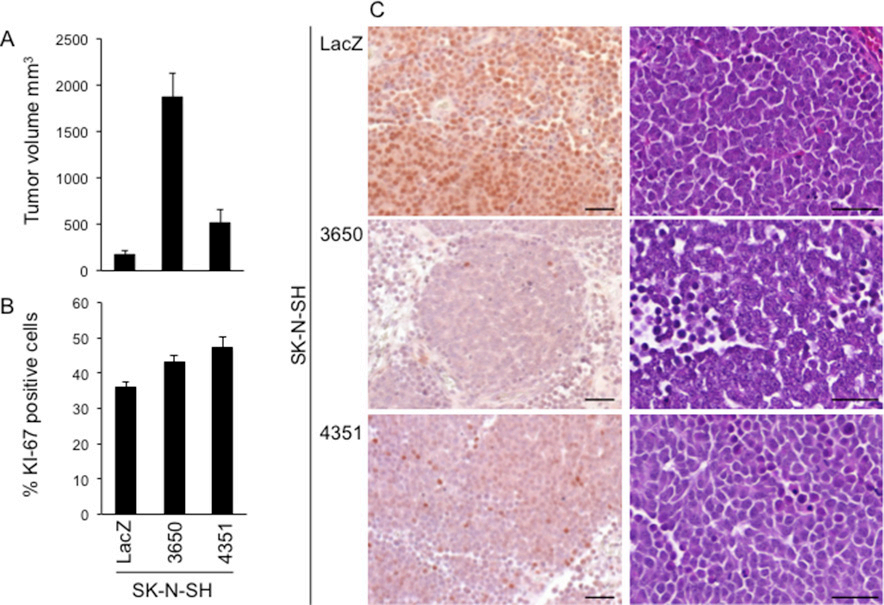
*ATM* silencing promotes SK-N-SH cell growth *in vivo* **A.** Five million SK-N-SH cells stably transfected with *ATM* shRNA vectors 3650 or 4351, or with LacZ shRNA vector as a control, were resuspended in 200 μl Matrigel and injected subcutaneously into the flank of 6-weeks old Swiss nu/nu female mice (5 mice/condition; 6 mice were used for SK-N-SH cells stably transfected with *ATM* shRNA vector 4351). The graph shows the tumor volume at the time of sacrifice ± SEM, 7 weeks after injection. *p* 3650 *vs* LacZ < 0.0001; *p* 4351 *vs* LacZ = 0.03 (*t*-test). **B.** Quantification of KI-67 immunostaining in the same tumors. The graph shows the percentage of KI-67 positive cells ± SEM from at least 400 counted cells from two different xenografts/condition. *p* 3650 *vs* LacZ = 0.005; *p* 4351 *vs* LacZ = 0.005 (two-tailed *t*-test) **C.** ATM immunohistochemistry (left column) or Hematoxylin-eosin staining (right column) of the indicated tumors. Bar = 50 μm.

To investigate the possibility that *ATM* silencing also confers a growth advantage to SK-N-SH cells *in vivo*, we injected SK-N-SH stable transfectants subcutaneously into Swiss *nu/nu* mice. In this model, SK-N-SH^kd-3650^ cells and SK-N-SH^kd-4351^ cells form tumors up to 10-fold larger than those formed by SK-N-SH^LacZ^ cells. Again, the effect was more marked with the SK-N-SH^kd-3650^ subline, having the strongest *ATM* silencing (Figure 7A, 5A). By immunohistochemistry, the levels of ATM expression were the highest in tumors formed by SK-N-SH^LacZ^ cells, the lowest in tumors formed by SK-N-SH^kd-3650^ cells, and intermediate in tumors formed by SK-N-SH^kd-4351^ cells, thus mirroring the expression pattern observed in the cell lines used for injection (Figure 7C left column; 5A). Histological analysis (hematoxylin and eosin staining, HE) revealed that all tumors formed by SK-N-SH cells, independently of *ATM* expression levels, were composed of sheets and nests of large cells, with large nuclei and prominent nucleoli, and scant to moderate amounts of cytoplasm, thus resembling undifferentiated large cell neuroblastoma [21] (Figure 7C right column). Consistent with the undifferentiated status of these tumors, the expression of several neuronal genes (*MAPT, GAP43, MAP2, APP, NEFL, NEFM, NEFH, NTRK2, SYP, TH*) [22–27] exhibits little or no differences in a cDNA microarray comparing SK-N-SH^kd-3650^ cells with SK-N-SH^LacZ^ cells, except that the expression of *NEFH* was 2.95-fold (mRNA) or 2.0-fold (protein) higher in SK-N-SH^kd-3650^ cells compared with SK-N-SH^LacZ^ cells. Expression of *NEFH* was not consistently upregulated in SK-N-SH^kd-4351^ cells that also have *ATM* silencing (Supplementary Figure S3; Figure 5A). Ki67 immunostaining revealed increased proliferation in xenografts carrying *ATM* silencing at the time of sacrifice (SK-N-SH^LacZ^ tumors: 36.03% ± 1.38; SK-N-SH^kd-3650^ tumors: 43.29% ± 1.61; SK-N-SH^kd-4351^ tumors: 47.09% ± 3.27; *p* SK-N-SH^kd-3650^ vs SK-N-SH^LacZ^ = 0.005; *p* SK-N-SH^kd-4351^ vs SK-N-SH^LacZ^ = 0.005; bilateral *t*-test) (Figure 7B). No metastases were observed in the three experimental groups at the time of dissection (brain, kidney, spleen, heart, liver, or lung) or in the subsequent histological analysis in the axillary or inguinal lymph nodes, heart, liver, or lung by HE staining.

In summary, these results demonstrate (*i*) that stable silencing of *ATM* confers a growth advantage to SK-N-SH cells *in vitro* and *in vivo*; (*ii*) that this effect is dependent on the extent of *ATM* silencing; and (*iii*) that partial silencing of *ATM* is sufficient to confer such growth advantage.

As a next step, we sought to determine if *ATM* silencing confers a growth advantage to additional NB cell lines. We silenced *ATM* expression in the CLB-Ga NB cell line by the same shRNA strategy. Like SK-N-SH cells, CLB-Ga cells have no *MYCN* amplification, wild type p53, and functional ATM [15–17].

**Figure 8:**
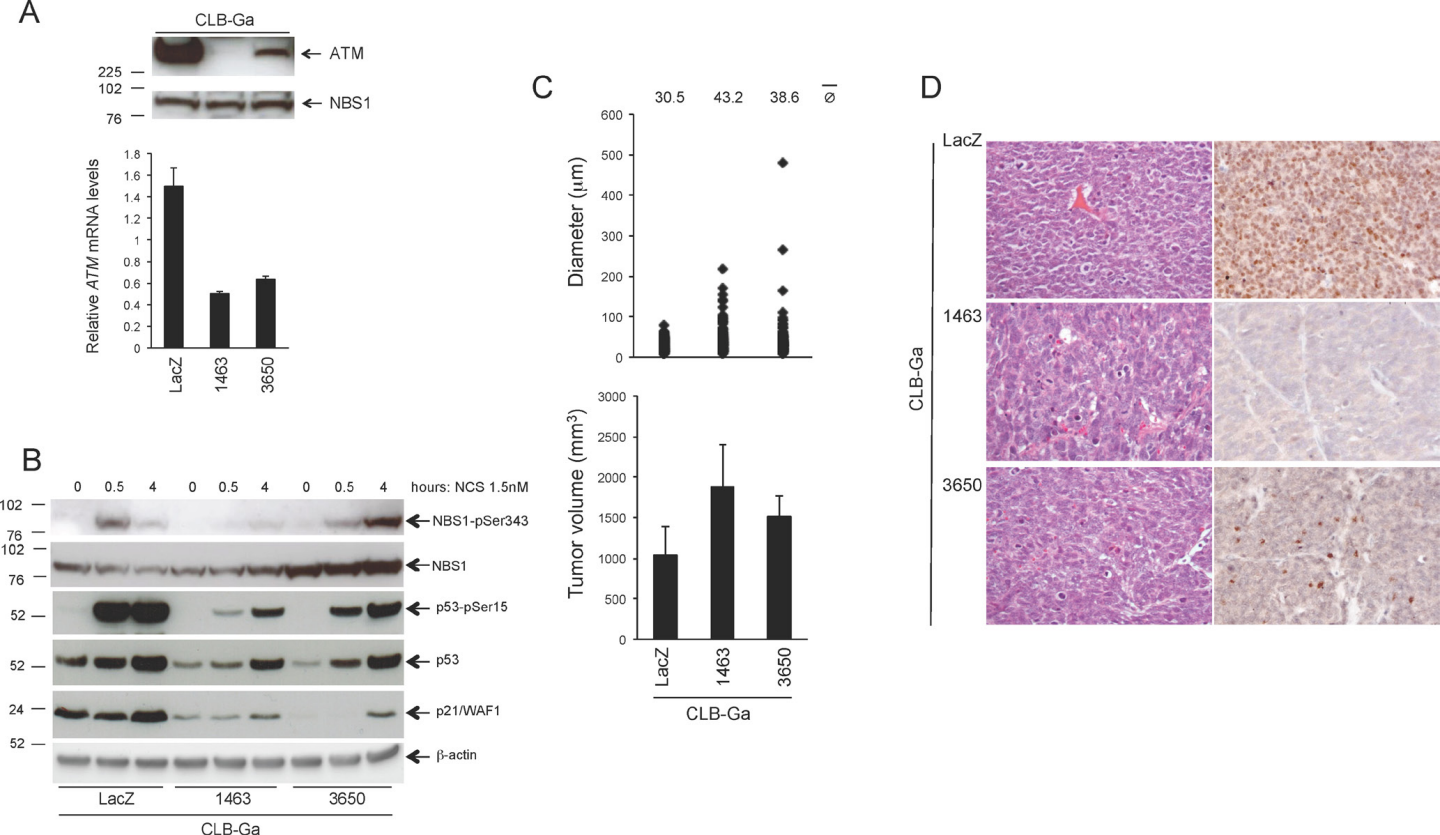
Phenotypic consequences of stable *ATM* silencing in CLB-Ga cells **A.** Western Blotting for ATM on CLB-Ga cells stably transfected with *ATM* shRNA vectors 1463, 3650 or with a LacZ shRNA vector as a control. NBS1 was used as a loading control. The graph (lower panel) shows the relative *ATM* mRNA levels as assessed by real-time quantitative PCR, ± SD from two different experiments. *p* 3650 *vs* LacZ < 0.001; *p* 1463 *vs* LacZ < 0.001 (two-tailed *t*-test) **B.** CLB-Ga cells stably transfected with *ATM* shRNA vectors 1463, 3650 or with a LacZ shRNA vector as a control were incubated in the presence of NCS 1.5 nM for the indicated time points and analyzed for NBS1-pSer343, NBS1, p53-pSer15, p53, p21/WAF1 or β-actin levels by Western Blotting. (**C**, upper panel) CLB-Ga cells stably transfected with *ATM* shRNA vectors 1463 or 3650, or with LacZ shRNA vector as a control, were resuspended in agarose gels at the density of 2 × 10^4^ cells/ml and grown for 14 days. The growth in agarose gels was quantified by measuring the diameter of the structures (single cells or multicellular colonies) formed after 14 days. At least 120 randomly selected structures per condition (single cells or multicellular colonies) from two independent experiments/condition were measured. *p* 1463 *vs* LacZ < 0.001; *p* 3650 *vs* LacZ < 0.05 (two-tailed *t*-test). **C.** lower panel Five million CLB-Ga cells stably transfected with *ATM* shRNA vectors 1463 or 3650, or with LacZ shRNA vector as a control, were resuspended in 200 μl Matrigel and injected subcutaneously into the flank of 6-weeks old Swiss nu/nu female mice (5 mice/condition). The graph shows the tumor volume at the time of sacrifice ± SEM, 4 weeks after injection. **D.** Hematoxylin-eosin staining (left) or ATM immunohistochemistry (right) of the indicated tumors. Bar = 20 μm.

Experiments with CLB-Ga cells included a third shRNA against *ATM* (1463). CLB-Ga cells stably expressing this shRNA (referred to as CLB-Ga^kd-1463^) had the strongest silencing of *ATM*, whereas CLB-Ga cells expressing *ATM* shRNA 3650 (referred to as CLB-Ga^kd-3650^) had an intermediate level of *ATM* silencing as assessed by Western blotting (Figure 8A, upper panel) and real-time quantitative PCR (Figure 8A, lower panel). Similar to SK-N-SH cells, phosphorylation of NBS1-Ser343 or p53-Ser15 and p21/WAF1 induction were reduced or delayed in CLB-Ga^kd-1463^ and CLB-Ga^kd-3650^ cells in response to NCS, compared to CLB-Ga^LacZ^ cells (Figure 8B). Similar to SK-N-SH cells, *ATM* silencing confers a growth advantage to CLB-Ga cells *in vitro* and *in vivo* (Figure 8C) although the *in vivo* effect was less marked compared to that observed in SK-N-SH cells (Figure 8C, lower panel). As seen in SK-N-SH cells, the *in vitro* and *in vivo* effect of *ATM* silencing was more marked in the subline with the most pronounced *ATM* silencing (CLB-Ga^kd-1463^; Figure 8A, 8C). Tumor histology (HE staining) was similar in the three experimental groups, all the tumors being densely cellular and highly undifferentiated, independent of *ATM* silencing (Figure 8D, left panel). No metastases were observed in the three experimental groups at the time of dissection or in the subsequent histological analysis (HE staining) in the brain, kidney, spleen, heart, liver, lung, lymph nodes or femurs.

Taken together, these results demonstrate that stable silencing of *ATM* confers an *in vitro* and *in vivo* growth advantage to two different NB cell lines characterized by normal *MYCN* status and functional ATM/p53. In both cell lines, partial silencing of *ATM* was sufficient to observe this effect.

### The tumor promoting effect of *ATM* silencing is *MYCN* independent

As potential mechanisms for the growth advantage observed in SK-N-SH and CLB-Ga cells with stable *ATM* silencing, we considered several possibilities.

First, we measured the basal levels of cell death in SK-N-SH^LacZ^, SK-N-SH^kd-3650^ and SK-N-SH^kd-4351^ cell cultures. Annexin V/7-Amino-Actinomycin (7-AAD) staining revealed similar levels of apoptosis in the three cell populations (SK-N-SH^LacZ^ : 5.46% ± 1.28; SK-N-SH^kd-3650^: 5.91% ± 1.62; SK-N-SH^kd-4351^: 6.47% ± 1.05; errors indicate SEM; *n* = 3). These results were corroborated by the quantification of the Sub-G1 cell population in cell cycle analyses (SK-N-SH^LacZ^: 1.14% ± 0.54; SK-N-SH^kd-3650^: 0.85% ± 0.32; SK-N-SH^kd-4351^: 1.73% ± 1.46; errors indicate SD; *n* = 2). Similar, low levels of cell death were observed when measuring the Sub-G1 cell fraction in CLB-Ga stable transfectants (CLB-Ga^LacZ^: 0.51% ± 0.20; CLB-Ga^−1463^: 1.15% ± 0.48; CLB-Ga^kd-3650^: 1.32% ± 1.24; errors indicate SD; *n* = 2) and in GI-ME-N stable transfectants (see below) (GI-ME-N^LacZ^: 2.01% ± 0.07; GI-ME-N^kd-1463^: 1.30% ± 0.85; GI-ME-N^kd-3650^: 2.89% ± 1.16; errors represent SD, *n* = 2).

**Figure 9:**
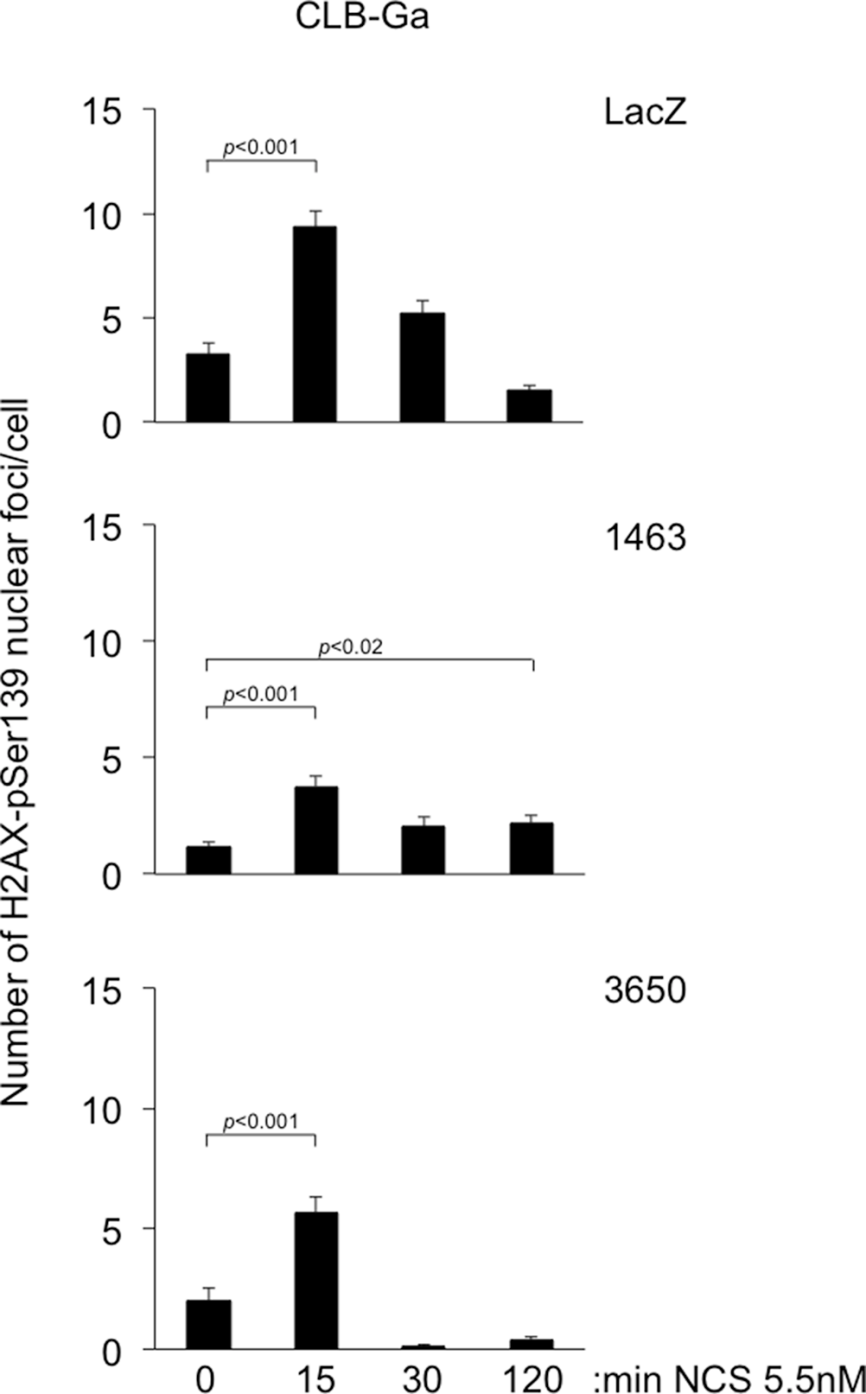
Induction and repair of H2AX-pSer139 nuclear foci in NCS treated CLB-Ga stable transfectants Approximately 70% confluent cultures of CLB-Ga cells stably transfected with *ATM* shRNA vectors 1463, 3650 or with a LacZ shRNA vector as a control were stained for H2AX-pSer139 nuclear foci at the indicated times after treatment with NCS 5.5 nM. The graphs show the mean number of H2AX-pSer139 nuclear foci/cell ± SEM from at least 40 counted cells/condition. *p* values in the Figure refer to two-tailed *t*-test.

Second, since defective DNA repair resulting from *ATM* silencing could generate mutations conferring a proliferative advantage, we analyzed the capacity of SK-N-SH and CLB-Ga stable transfectants to resolve histone H2AX-pSer139 nuclear foci in response to NCS. The induction and decay of H2AX-pSer139 nuclear foci in response to low doses of external mutagens is one of the most reliable and best characterized quantitative DNA repair assays [28]. SK-N-SH^kd-3650^ and CLB-Ga^kd-1463^ stable transfectants, having strong *ATM* silencing resulting in almost undetectable protein (Figure 5A, 8A), exhibit defective repair of NCS induced H2AX-pSer139 nuclear foci, consistent with previous results on *ATM* deficient cells [29, 30]. In contrast, partial silencing of *ATM* allows SK-N-SH^kd-4351^ cells and CLB-Ga^kd-3650^ cells to resolve the NCS induced H2AX-pSer139 nuclear foci with a kinetics comparable to the respective controls. Examples of the results obtained are shown in Figure 9 and Supplementary Figure S4. We conclude that the growth advantage observed in SK-N-SH and CLB-Ga cells with stable *ATM* silencing is not necessarily associated with a gross defect in DSB repair.

To further investigate the mechanism by which stable *ATM* silencing promotes NB progression, we performed two sets of experiments.

**Figure 10:**
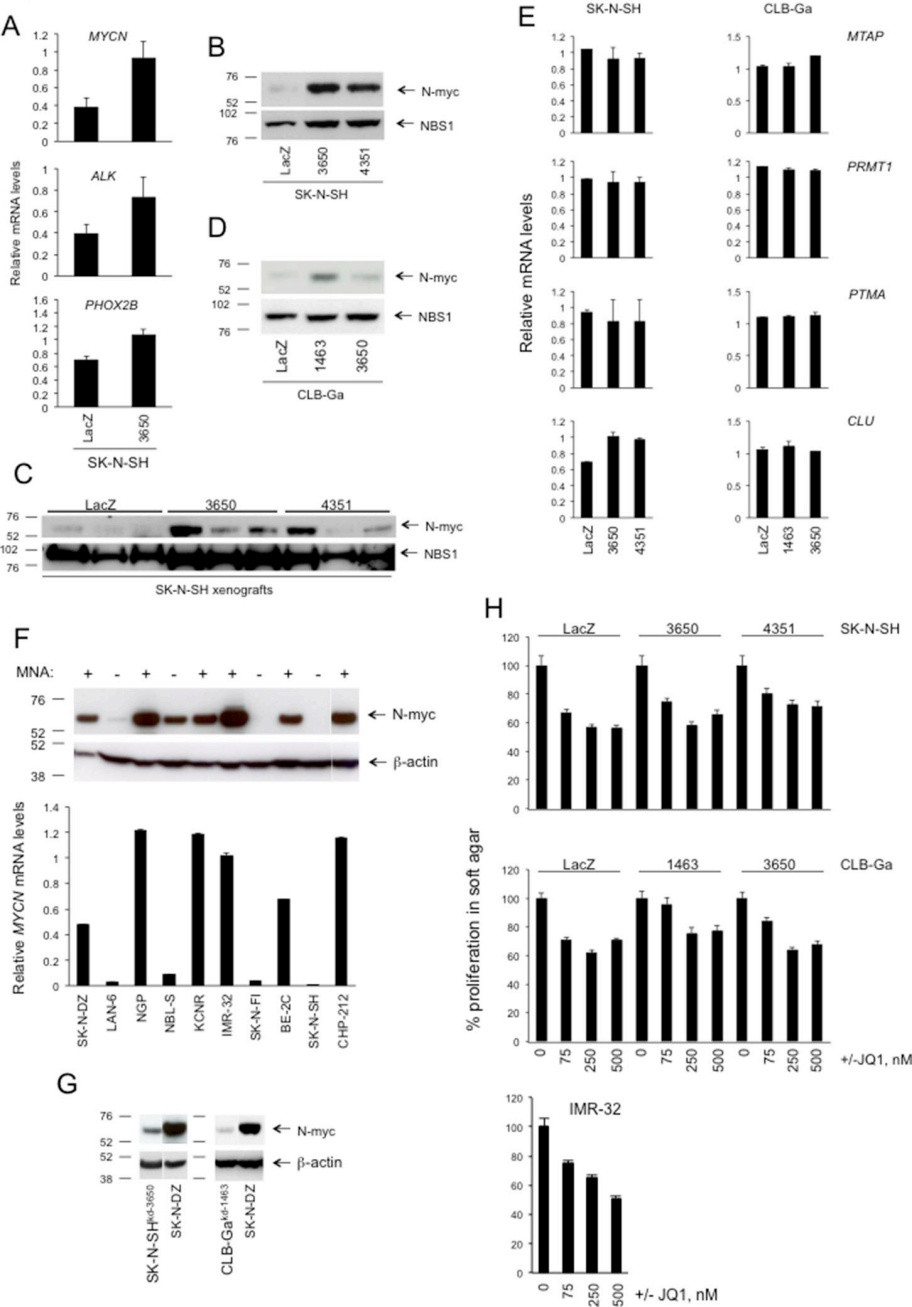
The effects of *ATM* silencing on SK-N-SH or CLB-Ga cells do not appear to require *MYCN* **A.** Relative mRNA levels for *MYCN*, *ALK*, *PHOX2B* in SK-N-SH cells stably transfected with *ATM* shRNA vectors 3650 or with LacZ shRNA vector. The graph shows the normalized values from real-time quantitative PCR analyses ± SEM from three different experiments. *p* values were as follows. *MYCN*: 3650 vs LacZ = 0.05; *ALK*: 3650 vs LacZ = 0.14; *PHOX2B*: 3650 vs LacZ = 0.02 (two-tailed *t*-test). **B.** Western Blotting for N-myc or NBS1 in SK-N-SH cells stably transfected with *ATM* shRNA vectors 3650, 4351 or with LacZ shRNA vector, or **C.** from tumors formed by these cells in nude mice (same tumors as Figure 7). **D.** Western Blotting for N-myc or NBS1 in CLB-Ga cells stably transfected with *ATM* shRNA vectors 1463, 3650 or with LacZ shRNA vector. **E.** The graphs show the normalized relative mRNA levels ± SD from two different experiments of the indicated *MYCN* target genes, as assessed by real-time quantitative PCR, in SK-N-SH cells or CLB-Ga cells stably transfected with *ATM* shRNA vectors 1463, 3650, 4351 or with LacZ shRNA vector. **F.** The indicated NB cell lines were analyzed for N-myc expression by Western Blotting (upper panel) or by real-time quantitative PCR (lower panel). In the graph, error bars represent SEM from internal replicates. For each cell line, the *MYCN* amplification (MNA) status is indicated. **G.** Western Blotting comparing the levels of N-myc between the SK-N-DZ cell line and SK-N-SH or CLB-Ga cells stably transfected with ATM shRNA vectors 3650 or 1463, respectively. **H.** SK-N-SH or CLB-Ga cells stably transfected with *ATM* shRNA vectors 1463, 3650 or 4351, or with LacZ shRNA vector, or the *MYCN* amplified IMR-32 neuroblastoma cell line were resuspended in agarose gels at the density of 2 × 10^4^ cells/ml and grown for 14 days in the presence or absence of the indicated concentrations of the N-myc inhibitor (+)-JQ1. The growth in agarose gels was quantified by measuring the diameter of the structures (single cells or multicellular colonies) formed after 14 days. At least 50 randomly selected structures per condition (single cells or multicellular colonies) were measured. Error bars represent SEM. The growth observed in the absence of (+)-JQ1 was set to 100% in order to facilitate the comparison among the different stable transfectants.

In the first set of experiments we looked at the expression of genes known to play an important role in the initiation or progression of NB. We selected *MYCN*, *ALK*, and *PHOX2B*. By real-time quantitative PCR, the three genes were upregulated in SK-N-SH^kd-3650^ cells compared to SK-N-SH^LacZ^cells, *MYCN* mRNA exhibiting the highest upregulation (2.5-fold). *ALK* and *PHOX2B* mRNAs were upregulated to a lesser extent (1.8-fold and 1.5-fold, respectively). Upregulation of *ALK* was not statistically significant (Figure 10A). For these reasons, we decided to investigate *MYCN* expression and transcriptional activity in more detail.

At the protein level, N-myc was upregulated by 6-fold in SK-N-SH^kd-3650^ cells compared to SK-N-SH^LacZ^ cells as assessed by scanning densitometry. N-myc was also upregulated in SK-N-SH^kd-4351^ cells, carrying a different *ATM* shRNA plasmid, thus confirming the specificity of this effect (Figure 10B). N-myc was also upregulated in tumors formed by SK-N-SH^kd-3650^ cells and SK-N-SH^kd-4351^ cells, compared to those formed by SK-N-SH^LacZ^ cells (Figure 10C). Similar but weaker upregulation of N-myc was also observed in CLB-Ga^kd-1463^ and CLB-Ga^kd-3650^ cells compared to CLB-Ga^LacZ^ cells (Figure 10D). No amplification of *MYCN* was observed in SK-N-SH^kd-3650^, SK-N-SH^kd-4351^, CLB-Ga^kd-1463^ or CLB-Ga^kd-3650^ cells by real-time quantitative PCR of genomic DNA, thus indicating that increased expression of *MYCN* in SK-N-SH and CLB-Ga cells with stable *ATM* silencing was not due to *MYCN* gene amplification (data not shown).

We then looked at *MYCN* transcriptional activity through the analysis of the expression of known *MYCN* regulated genes. For this analysis, we selected six genes (*ATAD2, CRTAP, DKC1, MTAP, PRMT1, PTMA*) known to be upregulated by *MYCN*, and two genes (*CLU, CNTN1*) known to be downregulated by *MYCN* [5, 31]. When compared to their respective controls (SK-N-SH^LacZ^ or CLBGa^LacZ^ cells), SK-N-SH^kd-3650^ or SK-N-SH^kd-4351^ cells, and CLB-Ga^kd-1463^ or CLB-Ga^kd-3650^ cells, despite having higher levels of N-myc expression (Figure 10A–10D), did not exhibit consistent corresponding regulation of the selected *MYCN* target genes (Figure 10E provides an example of the results obtained). To further investigate the possibility that N-myc upregulation observed in SK-N-SH and CLB-Ga cells with stable *ATM* silencing results in a *MYCN* transcriptional signature, we analyzed the 157 *MYCN* target gene signature [5] in our cDNA microarray comparing SK-N-SH^kd-3650^ cells with SK-N-SH^LacZ^ cells. Of the 157 *MYCN* regulated genes reported [5], 154 were present in our microarray (the 3 absent genes being: *C1orf97, FAM85A and LOC442075*). Of these, 138 genes exhibit a fold change ≤ ± 1.5. Ten genes (*CNTN1, MBNL2, RAD51AP1, RAD54L, RHOC, SESTD1, SKA3, SYNPO2, TP53INP1, UBE2H*) exhibit a fold change > ± 1.5 and ≤ ± 2.0. Six genes (*CLU, DNER, PDE5A, SUSD5, SYT4, TEX15*) exhibit a fold change > ± 2 and ≤ ± 7.99, with only two genes, *DNER* (downregulated 6.38-fold in SK-N-SH^kd-3650^ cells *vs* SK-N-SH^LacZ^ cells; thus consistent with negative regulation by *MYCN* [5]) and *TEX15* (upregulated 2.39-fold in SK-N-SH^kd-3650^ cells *vs* SK-N-SH^LacZ^ cells; thus consistent with positive regulation by *MYCN* [5]) presenting with a statistically significant regulation (*p* < 0.05, ANOVA).

Subsequent validation of *DNER* or *TEX15* by real-time quantitative PCR confirmed regulation of these two genes by *ATM* silencing in SK-N-SH^kd-3650^ cells *vs* SK-N-SH^LacZ^ cells but not in SK-N-SH^kd-4351^ cells or CLB-Ga stable transfectants (data not shown). Similar results were obtained with miR-19a-5p, a member of the miR-17-92 cluster, known to be upregulated by *MYC/MYCN* [32], whereas another member of the miR-17-92 cluster, namely miR-92a-1-5p, or *DKK3*, another *MYCN* regulated gene [33] were not regulated by *ATM* silencing in SK-N-SH or CLB-Ga cells as assessed by real-time quantitative PCR (Supplementary Figure S5).

Taken together, our results indicate that although very limited and weak signs of *MYCN* target gene regulation are observed in SK-N-SH cells with stable *ATM* silencing, such signs are limited to a very small fraction of known *MYCN* regulated genes and are not observed across all the SK-N-SH or CLB-Ga cells with stable *ATM* silencing considered.

To investigate the possibility that this depends on the level of N-myc expression, we compared the level of N-myc expressed by SK-N-SH^kd-3650^ cells to that expressed by a NB cell line known to carry *MYCN* amplification. For this analysis we chose the SK-N-DZ cell line, which is the one with the lowest level of N-myc protein expression, among the ones available in our laboratory (Figure 10F). Compared to SK-N-DZ cells, SK-N-SH^kd-3650^ cells or CLB-Ga^kd-1463^ cells have an approximately 20-fold lower level of N-myc as assessed by scanning densitometry (Figure 10G). These results suggest that, although *ATM* silencing consistently upregulates N-myc expression in SK-N-SH and CLB-Ga cells, this level of N-myc, or the entity of the upregulation, is not sufficient to trigger a typical *MYCN* target gene signature.

In the second set of experiments, we extended our analysis to the whole transcriptome of SK-N-SH^kd-3650^ and SK-N-SH^LacZ^ cells by cDNA microarray. Thirteen genes were found up- or downregulated by at least 2-fold (*p* < 0.01) in SK-N-SH^kd-3650^ cells compared to SK-N-SH^LacZ^ cells (*COX17, TSTD1, ACOT1, ACOT2, MYD88, B2GALNT1, CDO1, SHC1, ARMCX1, ZNF208, MAGEC2, BCHE, IMPA2*). However, subsequent real-time quantitative validation of this mRNA signature in SK-N-SH^kd-4351^, CLB-Ga^LacZ^, CLB-Ga^kd-1463^, and CLB-Ga^kd-3650^ cells did not confirm consistent regulation of these genes by *ATM* silencing across these different SK-N-SH or CLB-Ga stable transfectants.

As a next step, we performed experiments aiming at demonstrating more directly that the tumor promoting effect of *ATM* silencing is not mediated by *MYCN*.

First, we assessed the sensitivity of SK-N-SH or CLB-Ga stable transfectant proliferation toward (+)-JQ1, a specific inhibitor of *MYCN* transcription and transcriptional activity that more markedly inhibits proliferation in NB cells with *MYCN* amplification than in NB cells without *MYCN* amplification [34]. In the soft agar assay, (+)-JQ1 inhibited cellular proliferation in SK-N-SH and CLB-Ga stable transfectants with similar efficiency, independently of *ATM* silencing (Figure 10H).

Second, we silenced the expression of *ATM* in the GI-ME-N cell line. Similar to SK-N-SH and CLB-Ga cells, GI-ME-N cells have no amplification of *MYCN* and retain a functional ATM/p53 response to DNA damage [17, 35]. In addition, these cells have no detectable *MYCN* expression as assessed by Western Blotting and real-time quantitative PCR (ref. [35]; and see below).

**Figure 11:**
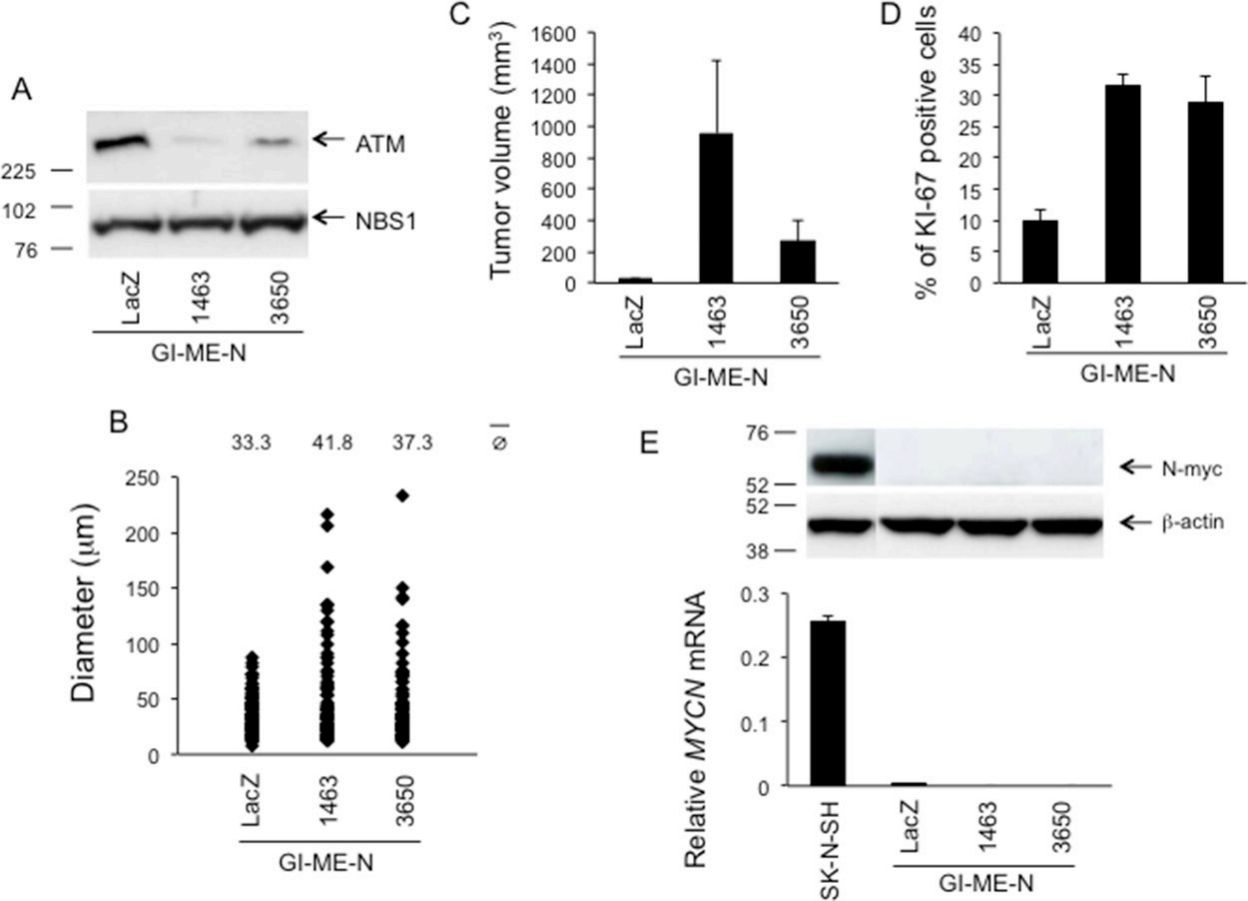
Phenotypic consequences of stable *ATM* silencing in GI-ME-N cells **A.** Western Blotting for ATM on GI-ME-N cells stably transfected with *ATM* shRNA vectors 1463, 3650 or with a LacZ shRNA vector as a control. NBS1 was used as a loading control. **B.** GI-ME-N cells stably transfected with *ATM* shRNA vectors 1463 or 3650, or with LacZ shRNA vector as a control, were resuspended in agarose gels at the density of 2 × 10^4^ cells/ml and grown for 14 days. The growth in agarose gels was quantified by measuring the diameter of the structures (single cells or multicellular colonies) formed after 14 days. At least 120 randomly selected structures per condition (single cells or multicellular colonies) from two independent experiments/condition were measured. *p* 1463 *vs* LacZ = 0.01; *p* 3650 *vs* LacZ = 0.04 (two-tailed *t*-test). **C.** Five million GI-ME-N cells stably transfected with *ATM* shRNA vectors 1463 or 3650, or with LacZ shRNA vector as a control, were resuspended in 200 μl Matrigel and injected subcutaneously into the flank of 6-weeks old Swiss nu/nu female mice (5 mice/condition). The graph shows the tumor volume at the time of sacrifice ± SEM, 12 weeks after injection. **D.** Quantification of KI-67 immunostaining of the tumors shown in C. The graph shows the percentage of KI-67 positive cells ± SEM from at least 600 counted cells from two different xenografts/condition. *p* 1463 *vs* LacZ < 0.01; *p* 3650 *vs* LacZ < 0.01 (two-tailed *t*-test). **E.** Analysis of *MYCN* expression at the mRNA (lower panel) level by real-time quantitative PCR (± SD from two different experiments) or at the protein (upper panel) level by Western Blotting on SK-N-SH cells, or GI-ME-N cells stably transfected with *ATM* shRNA vectors no. 1463, no. 3650 or with a LacZ shRNA vector as a control.

GI-ME-N stable transfectants expressing *ATM* shRNA 1463 (referred to as GI-ME-N^kd-1463^ cells) have a 6-fold reduction in ATM protein expression compared to GI-ME-N cells expressing a LacZ control shRNA (referred to as GI-ME-N^LacZ^ cells) whereas GI-ME-N cells expressing *ATM* shRNA 3650 (referred to as GI-ME-N^kd-3650^) exhibit an intermediate silencing of *ATM* (Figure 11A). Similar to SK-N-SH and CLB-Ga cells, phosphorylation of p53-Ser15 and p21/WAF1 induction were reduced or delayed in GI-ME-N^kd-1463^ and GI-ME-N^kd-3650^ cells in response to NCS compared to GI-ME-N^kd-LacZ^ cells (Supplementary Figure S6). In the soft agar assay, GI-ME-N^kd-1463^ and GI-ME-N^kd-3650^ cells form colonies larger than those formed by GI-ME-N^LacZ^ cells. Again, the effect was more marked in the subline having the strongest silencing of *ATM* (GI-ME-N^kd-1463^) (Figure 11B). When injected into nude mice, GI-ME-N^kd-1463^ and GI-ME-N^kd-3650^ cells formed tumors 33.6- and 9.3-fold larger, respectively, than those formed by GI-ME-N^kd-LacZ^ cells (Figure 11C). Ki67 immunostaining revealed increased proliferation in xenografts carrying *ATM* silencing at the time of sacrifice (Figure 11D). No distant metastases were observed at the time of dissection or in the subsequent histological analysis (HE staining) in the brain, kidney, spleen, heart, liver, lung, lymph nodes or femurs of mice injected with GI-ME-N^kd-1463^, GI-ME-N^kd-3650^ or GI-ME-N^LacZ^ cells. *MYCN* expression was undetectable in GI-ME-N^kd-3650^, GI-ME-N^kd-1463^ and GI-ME-N^kd-LacZ^ cells by Western Blotting and real-time quantitative PCR (Figure 11E), thus further corroborating the conclusion that the tumor promoting effect of *ATM* silencing in the NB cell background does not require *MYCN*.

In summary, our results show (*i*) that *ATM* is frequently deleted in NB, and that deletion correlates with lower *ATM* expression, EFS and OS; (*ii*) that stable silencing of *ATM* by three different *ATM* shRNAs confers a growth advantage to three different NB cell lines *in vitro* and *in vivo*; (*iii*) that this effect is dependent on the extent of ATM silencing; (*iv*) that partial silencing of *ATM* is sufficient to observe such growth advantage, and (*v*) that this effect does not require the expression or the activity of *MYCN*.

## DISCUSSION

The proportion of NB with 11q deletion is low in forms having a more favorable prognosis (localized and 4S stages), which represent 8 to 21% of NB cases. However, it is significantly higher in aggressive metastatic stage 4, where this alteration is observed in more than half of the tumors [2, 36]. Several studies reported an association between 11q aberrations and unfavorable outcome [2, 36–39] thus suggesting that patients presenting with localized and 4S stages NB and 11q deletion would benefit from a more intensive therapy. On this background, 11q deletion was recently added in the INRG classification as an independent prognostic marker, predicting poor outcome in a subset of cases with intermediate risk [2, 36, 40]. The importance of 11q deletion as an independent prognostic marker is further highlighted by its capacity to predict poor prognosis in NB devoid of a typical *MYCN* target gene signature due to *MYCN* amplification or N-myc protein stabilization (ref. [5]; this paper). An intriguing finding is the inverse correlation of *MYCN* amplification and 11q deletion, with very infrequent cases described carrying both cytogenetic alterations. These cases are characterized by a dramatic decline of survival rates [36].

In addition to its prognostic value, 11q deletion might have functional implications in NB, possibly through the haploinsufficiency of tumor suppressor(s) contributing to the progression of this disease. Usually, 11q deletion involves a large distal part of the chromosome 11 spanning over 60 Mb from 11q13 to 11qter [2], but smaller deletions were also identified, leading to the identification of a shortest region of overlap (SRO). Different SRO were reported in sporadic NB, mostly at 11q23 (11q23.3 [37]; 11q14-q23 [41]; 11q23.3-q25 [42]), but no consensus region was found suggesting that there may be more than one 11q NB suppressor gene [43]. Other 11q NB suppressor genes might be *TSLC1* (also named *CADM1* or *IGSF4*), *SDHD* and *H2AX* [44–48].

Chromosome 11q deletion has been suggested to occur late after NB initiation [49] thus raising the possibility that it might contribute to the late phases of NB progression. If this hypothesis were confirmed by future data, it might explain why AT patients or carriers have not been reported to be at higher risk for NB. Well-established tumor suppressors are frequently mutated in somatic cancers which are not or rarely associated with germline mutations in the same genes. This is the case for *TP53*, mutated in approximately 60% of sporadic lung, ovary, bladder, intestine, and head and neck cancers, whose frequency exhibits little or no increase in the Li Fraumeni syndrome [50–52], or for *ATM* itself, frequently mutated in sporadic colon cancer (cancer.sanger.ac.uk/cancergenome/projects/cosmic/), not a typical hallmark of AT [7]. In these settings, *ATM* or *TP53* inactivation is likely to contribute to the progression rather than to the initiation of these tumors. Initiating events in NB might include the gain of 17q, the most frequent genetic abnormality in NB (ref. [53], and references therein). Along the same lines, it is interesting to note that *TP53*, which works downstream of *ATM*, is rarely mutated in primary NB. Consistent with this notion, NB is rare in Li Fraumeni syndrome. However, *TP53* is frequently mutated in NB relapses [16, 54, 55].

Based on our epidemiological data showing that reduced expression due to haploinsufficiency, rather than mutation, affects *ATM* status in NB, we generated NB cell lines with stable *ATM* silencing to investigate the possibility that reduction of *ATM* expression due to 11q deletion might contribute to NB progression. In the three NB cell lines considered, *ATM* silencing confers an *in vitro* and *in vivo* growth advantage as assessed on the basis of the soft agar assay and subcutaneous xenografts in nude mice. This effect could be due in part to the reduced basal levels of p21/WAF1 expression, consistently observed in the three NB cell lines with stable *ATM* silencing generated. In our model, *ATM* silencing does not seem to confer metastatic capacity to NB cells. However, metastasis of human cells in nude mice is infrequent. The consistency of our data across three different NB cell lines, the tumor progression effect resulting from partial *ATM* silencing and the *MYCN* independence of the phenotype observed suggest that 11q deletion is causally involved in NB progression and that *ATM* haploinsufficiency contributes to this effect.

These results provide experimental evidence that *ATM* deletion, an event frequently observed in NB with poor prognosis and not carrying *MYCN* amplification, might be causally involved in the progression of this NB tumor subset. From a clinical point of view, these data suggest that activation of branches of the ATM pathway by small molecule drugs (e.g. the p53 activator nutlin-3 [16, 56]) might be of benefit to patients carrying this particular subset of NB. Since 11q also carry additional genes regulating the DNA damage response (e.g. *MRE11*, *H2AX*) it is possible that simultaneous deletion of these genes contributes individually to the aggressive behavior of NB carrying 11q deletion, and that the poor prognosis of NB carrying 11q deletion results from the simultaneous inactivation of several tumor suppressors, *ATM* being one of them.

## MATERIALS AND METHODS

### Ethics statement

Investigation has been conducted in accordance with the ethical standards and according to the Declaration of Helsinki and according to national and international guidelines and has been approved by the authors' institutional review board.

### NB tumor sets

We used two distinct NB tumor sets. Fifty NB DNA samples (NB50) collected from children who, or their representatives, had consented to anonymous use in research were obtained from the Children's Oncology Group Neuroblastoma Biology Committee. Twelve were stage 1, 8 were stage 2, 6 were stage 3 and 24 were stage 4 NB. DNA from 60 Caucasian blood donors without family history of cancer was used as control. The NB110 set is an extended set of NB88 [5] with samples from patients with NB of all stages. Written informed consent was obtained from patients' parents in accordance with review board policies and procedures for research dealing with tumor specimen and clinical information. The medical ethics committee of the Academic Medical Center (AMC) in Amsterdam approved the study. All NB samples were derived from primary tumors of untreated patients. mRNA was isolated and analyzed using Affymetrix (Santa Clara, CA) Human Genome U133 Plus 2.0 arrays and were normalized using MAS5.0 (accession no. GSE16476). Genomic aberrations were scored by arrayCGH and/or cgCGH (coverage profiles of complete genomics sequencing data resulting in CGH-like plots [12]). We used a cut-off of −0.5 (2log) to score *ATM* loss. Data were analyzed using the R2 platform (http://r2.amc.nl).

### Analysis of *ATM* alterations in NB samples and NB cell lines

We analyzed the DNA of 16 NB cell lines by standard FISH using a commercial *ATM* probe (Abbott, Baar, Switzerland). The 66 coding exons of the *ATM* gene were screened for mutations by DHPLC (Wave™ System, Transgenomic Inc., San Jose, CA, USA). Alterations detected by DHPLC were analyzed by direct sequencing [57]. To determine the relative copy number of the *ATM* exons, we analyzed large gene rearrangements by Multiplex Ligation-dependent Probe Amplification assay (MLPA; SALSA P041 and SALSA P042 ATM, MRC-Holland, The Netherlands) according to manufacturer's instructions and as reported [58]. *ATM* methylation status was assessed by methylation specific MLPA assay (MS-MLPA, ME001B Tumor suppressor-1 and ME002 Tumor suppressor, MRC-Holland), according to manufacturer's instructions and as reported [59].

### Statistical considerations

For tests of association and survival analyses within the NB50 series, clinical data of 49 patients were included (one patient was excluded because of *ATM* duplication). For EFS, time to event was calculated from diagnosis until the first occurrence of relapse, progression, secondary malignancy, or death from any cause, or until last contact if no event occurred. For OS, time from diagnosis to death was calculated, or until last contact if the patient was alive. Curves were compared using a logrank test.

In the NB110 series, EFS was calculated in the same way as in the NB50 series, the only difference being that those events where the ‘death reason’ was specified as being a toxic death were removed. Cox regression analyses were performed in R, using the coxph() function of the survival library with either 1 or 2 covariates.

### Cell culture

NB cell lines were cultured as described [59]. The soft agar assay was performed as described [14].

### *ATM* silencing

Stable silencing of *ATM* was achieved by stably transfecting NB cell lines with pSuper-neo (Oligoengine, Seattle, Washington, USA) expressing shRNA targeting LacZ mRNA (CGACUACACAAAU CAGCGA) or three different *ATM* mRNA sequences (1463: GAUACCAGAUCCUUGGA GAU; 3650: GCUGCAGAGUCAAUCAAUAGA; 4351: GCAACAUUUGCCUAUAUCA). Stable transfectants were selected in G418 according to pSuper-neo manufacturer's instructions.

### Quantitative real-time PCR

Quantitative real-time PCR was performed as described [14]. Primer sequences were as follows:

*ATM*: TGCTGACAATCATCACCAAGTTC; TCTCCCTTCGTGTCCTGGAA
*MYCN*: CCTTCGGTCCAGCTTTCTCA; GCG GCCTTCTCATTCTTTACC
*ALK*: CCCCGCCTTCTCTTCCA; GGCATGT TTGTTGGTGATTCC
*PHOX2B*: GTCCGTACGCCGCAGTTC; GCTTG CGCTTCTCGTTGAG
*MTAP* : CCCCAAAACGAGAGAGGTTCT ; GCAC CGGAGTCCTAGCTTCTTA
*PRMT1*: CGCAAGGTCATCGGGATC; CTTCACC GCATAATCAGAGATACTG
*PTMA*: GATGACACGCGCTCTCCAC; CTGTTGC AAATTCTCATGGTTTG
*CLU*: TTGGCCGCCAGCTTGA; AGAAGT AGAAGGGCGAGCTCTG

### Western blotting

Western Blotting and densitometry for ATM, β-actin, NBS1, NBS1-pSer343 or N-myc were as described [14]. Western Blotting for N-myc, p53-pSer15, p53 or p21/WAF1 used antibody sc-53993 (Santa Cruz Biotechnology (Dallas, Texas, USA)), 9284 (Cell Signaling Technology), DO-1/sc-126 (Santa Cruz Biotechnology) or SX118/sc-53870 (Santa Cruz Biotechnology) respectively. Second antibody/HRP complexes were revealed with Roche Lumi-Light (Cat. No. 12015200001) or Lumi-Light Plus (Cat. No. 12015218001) Western Blotting Substrate, depending on the level of sensitivity required. Blots were scanned with a Hewlett-Packard 1536dnf scanner and imported using Microsoft Fax and Scan v. 6.1.

### Xenografts

Five million cells were resuspended in Growth Factor Reduced Matrigel (cat. No. 354230, VWR International GmbH, Dietikon, Switzerland) and injected subcutaneously into the flank of 6 weeks-old Swiss nude female mice (Charles River Laboratories, L'Arbresle Cedex, France). Five mice/condition were used. Mice were maintained in a specific pathogen free facility. Experiments were performed according to Institutional ethics guidelines and to the Swiss law.

### ATM and Ki-67 immunohistochemistry

FFPE Tissue sections were deparaffinized and rehydrated using standard procedures. Heat-induced epitope retrieval was carried out in a BioCare Medical (Concord, CA, USA) pressure cooker (Decloaking ChamberTM NxGen) with 10mM citrate buffer (pH 6.0) at 110°C for 12 min. Samples were incubated with a rabbit anti-Ki67 antibody (ab16667, Abcam, UK, diluted 1:100), or a rabbit anti-ATM antibody (ab32420, Abcam, UK, diluted 1:200) respectively 1 hour at RT or overnight at 4°C in a moist chamber. The signal was revealed with MACH 4 Universal HRP-Polymer Detection Kit (BioCare) according to the manufacturer's instructions. Purified rabbit IgG (02–6102, Life Technologies, Switzerland) was used as a negative control. Stained sections were photographed with a Zeiss Axio Vert.A1 microscope. Images were imported with AxioVision v. 4.8.2.0.

### Flow cytometry

Apoptosis was measured by flow cytometry using the Annexin V apoptosis detection Kit (BD Pharmingen cat. no. 556547) according to manufacturer's instructions. Cell cycle analysis used the BD Pharmingen BrdU Flow Kit (cat. no. 552598). Cells were labelled with 20 μM 5-bromo-2′-deoxyuridine (BrdU) for 4 h. After BrdU incorporation, cells were stained according to manufacturer's protocol. BrdU was omitted in the negative control.

All the data were collected with a Beckman Coulter FACS CyAn analyzer. Analyses were performed with Kalusa software (Beckman Coulter).

### γ-H2AX immunofluorescence

Immunofluorescence with phospho-Ser139 H2AX antibody JBW301 (catalogue no. 05–636, Millipore, Zug, Switzerland) was performed as described [60].

## SUPPLEMENTARY FIGURES



## References

[1] Cheung NK, Dyer MA (2013). Neuroblastoma: developmental biology, cancer genomics and immunotherapy. Nat Rev Cancer.

[2] Attiyeh EF, London WB, Mossé YP, Wang Q, Winter C, Khazi D, McGrady PW, Seeger RC, Look AT, Shimada H, Brodeur GM, Cohn SL, Matthay KK (2005). Chromosome 1p and 11q deletions and outcome in neuroblastoma. N Engl J Med.

[3] Weiss WA, Aldape K, Mohapatra G, Feuerstein BG, Bishop JM (1997). Targeted expression of MYCN causes neuroblastoma in transgenic mice. EMBO J.

[4] Molenaar JJ, Domingo-Fernández R, Ebus ME, Lindner S, Koster J, Drabek K, Mestdagh P, van Sluis P, Valentijn LJ, van Nes J, Broekmans M, Haneveld F, Volckmann R (2012). LIN28B induces neuroblastoma and enhances MYCN levels via let-7 suppression. Nat Genet.

[5] Valentijn LJ, Koster J, Haneveld F, Aissa RA, van Sluis P, Broekmans ME, Molenaar JJ, van Nes J, Versteeg R (2012). Functional MYCN signature predicts outcome of neuroblastoma irrespective of MYCN amplification. Proc Natl Acad Sci USA.

[6] Ditch S, Paull TT (2012). The ATM protein kinase and cellular redox signaling: beyond the DNA damage response. Trends Biochem Sci.

[7] Perlman SL, Boder E, Sedgewick RP, Gatti RA (2012). Ataxia-telangiectasia. Handb Clin Neurol.

[8] Kuilman T, Michaloglou C, Mooi WJ, Peeper DS (2010). The essence of senescence. Genes Dev.

[9] Hu H, Du L, Nagabayashi G, Seeger RC, Gatti RA (2010). ATM is down-regulated by N-Myc-regulated microRNA-421. Proc Natl Acad Sci USA.

[10] Scott SP, Bendix R, Chen P, Clark R, Dork T, Lavin MF (2002). Missense mutations but not allelic variants alter the function of ATM by dominant interference in patients with breast cancer. Proc Natl Acad Sci USA.

[11] Pugh TJ, Morozova O, Attiyeh EF, Asgharzadeh S, Wei JS, Auclair D, Carter SL, Cibulskis K, Hanna M, Kiezun A, Kim J, Lawrence MS, Lichenstein L (2013). The genetic landscape of high-risk neuroblastoma. Nat Genet.

[12] Molenaar JJ, Koster J, Zwijnenburg DA, van Sluis P, Valentijn LJ, van der Ploeg I, Hamdi M, van Nes J, Westerman BA, van Arkel J, Ebus ME, Haneveld F, Lakeman A (2012). Sequencing of neuroblastoma identifies chromothripsis and defects in neuritogenesis genes. Nature.

[13] Sausen M, Leary RJ, Jones S, Wu J, Reynolds CP, Liu X, Blackford A, Parmigiani G, Diaz LA, Papadopoulos N, Vogelstein B, Kinzler KW, Velculescu VE (2013). Integrated genomic analyses identify ARID1A and ARID1B alterations in the childhood cancer neuroblastoma. Nat Genet.

[14] Mandriota SJ, Buser R, Lesne L, Stouder C, Favaudon V, Maechler P, Béna F, Clément V, Rüegg C, Montesano R, Sappino AP (2010). Ataxia telangiectasia mutated (ATM) inhibition transforms human mammary gland epithelial cells. J Biol Chem.

[15] Thiele CJ, Masters (1998). Neuroblastoma. J Human Cell Culture.

[16] Van Maerken T, Speleman F, Vermeulen J, Lambertz I, De Clercq S, De Smet E, Yigit N, Coppens V, Philippé J, De Paepe A, Marine JC, Vandesompele J (2006). Small-molecule MDM2 antagonists as a new therapy concept for neuroblastoma. Cancer Res.

[17] Mergui X, Leteurtre F, Lipinski M, Bénard J, Amor-Guéret M (2008). Two distinctly altered cellular responses to DNA double-strand breaks in human neuroblastoma. Biochimie.

[18] Shiloh Y, Ziv Y (2013). The ATM protein kinase: regulating the cellular response to genotoxic stress, and more. Nat Rev Mol Cell Biol.

[19] Favaudon V (1983). Gamma-radiolysis study of the reductive activation of neocarzinostatin by the carboxyl radical. Biochimie.

[20] el-Deiry WS, Tokino T, Velculescu VE, Levy DB, Parsons R, Trent JM, Lin D, Mercer WE, Kinzler KW, Vogelstein B (1993). WAF1, a potential mediator of p53 tumor suppression. Cell.

[21] Tornóczky T, Kálmán E, Kajtár PG, Nyári T, Pearson AD, Tweddle DA, Board J, Shimada H (2004). Large cell neuroblastoma: a distinct phenotype of neuroblastoma with aggressive clinical behavior. Cancer.

[22] Encinas M, Iglesias M, Liu Y, Wang H, Muhaisen A, Ceña V, Gallego C, Comella JX (2000). Sequential treatment of SH-SY5Y cells with retinoic acid and brain-derived neurotrophic factor gives rise to fully differentiated, neurotrophic factor-dependent, human neuron-like cells. J Neurochem.

[23] Ruiz-León Y, Pascual A (2001). Brain-derived neurotrophic factor stimulates beta-amyloid gene promoter activity by a Ras-dependent/AP-1-independent mechanism in SH-SY5Y neuroblastoma cells. J Neurochem.

[24] Ruiz-León Y, Pascual A (2003). Induction of tyrosine kinase receptor b by retinoic acid allows brain-derived neurotrophic factor-induced amyloid precursor protein gene expression in human SH-SY5Y neuroblastoma cells. Neuroscience.

[25] Irwin N, Chao S, Goritchenko L, Horiuchi A, Greengard P, Nairn AC, Benowitz LI (2002). Nerve growth factor controls GAP-43 mRNA stability via the phosphoprotein ARPP-19. Proc Natl Acad Sci U S A.

[26] Jin K, Mao XO, Batteur S, Sun Y, Greenberg DA (2003). Induction of neuronal markers in bone marrow cells: differential effects of growth factors and patterns of intracellular expression. Exp Neurol.

[27] Woodbury D, Reynolds K, Black IB (2002). Adult bone marrow stromal stem cells express germline, ectodermal, endodermal, and mesodermal genes prior to neurogenesis. J Neurosci Res.

[28] Rothkamm K, Löbrich M (2003). Evidence for a lack of DNA double-strand break repair in human cells exposed to very low x-ray doses. Proc Natl Acad Sci U S A.

[29] Riballo E, Kühne M, Rief N, Doherty A, Smith GC, Recio MJ, Reis C, Dahm K, Fricke A, Krempler A, Parker AR, Jackson SP, Gennery A (2004). A pathway of double-strand break rejoining dependent upon ATM, Artemis, and proteins locating to gamma-H2AX foci. Mol Cell.

[30] Kühne M, Riballo E, Rief N, Rothkamm K, Jeggo PA, Löbrich M (2004). A double-strand break repair defect in ATM-deficient cells contributes to radiosensitivity. Cancer Res.

[31] Lutz W, Stöhr M, Schürmann J, Wenzel A, Löhr A, Schwab M (1996). Conditional expression of N-myc in human neuroblastoma cells increases expression of alpha-prothymosin and ornithine decarboxylase and accelerates progression into S-phase early after mitogenic stimulation of quiescent cells. Oncogene.

[32] Mogilyansky E, Rigoutsos I (2013). The miR-17/92 cluster: a comprehensive update on its genomics, genetics, functions and increasingly important and numerous roles in health and disease. Cell Death Differ.

[33] Bell E, Lunec J, Tweddle DA (2007). Cell cycle regulation targets of MYCN identified by gene expression microarrays. Cell Cycle.

[34] Puissant A, Frumm SM, Alexe G, Bassil CF, Qi J, Chanthery YH, Nekritz EA, Zeid R, Gustafson WC, Greninger P, Garnett MJ, McDermott U, Benes CH (2013). Targeting MYCN in neuroblastoma by BET bromodomain inhibition. Cancer Discov.

[35] Cornaglia-Ferraris P, Ponzoni M, Montaldo P, Mariottini GL, Donti E, Di Martino D, Tonini GP (1990). A new human highly tumorigenic neuroblastoma cell line with undetectable expression of N-myc. Pediatr Res.

[36] Spitz R, Hero B, Simon T, Berthold F (2006). Loss in chromosome 11q identifies tumors with increased risk for metastatic relapses in localized and 4S neuroblastoma. Clin Cancer Res.

[37] Guo C, White PS, Weiss MJ, Hogarty MD, Thompson PM, Stram DO, Gerbing R, Matthay KK, Seeger RC, Brodeur GM, Maris JM (1999). Allelic deletion at 11q23 is common in MYCN single copy neuroblastomas. Oncogene.

[38] Luttikhuis ME, Powell JE, Rees SA, Genus T, Chughtai S, Ramani P, Mann JR, McConville CM (2001). Neuroblastomas with chromosome 11q loss and single copy MYCN comprise a biologically distinct group of tumours with adverse prognosis. Br J Cancer.

[39] Simon T, Spitz R, Hero B, Berthold F, Faldum A (2006). Risk estimation in localized unresectable single copy MYCN neuroblastoma by the status of chromosomes 1p and 11q. Cancer Lett.

[40] Cohn SL, Pearson AD, London WB, Monclair T, Ambros PF, Brodeur GM, Faldum A, Hero B, Iehara T, Machin D, Mosseri V, Simon T, Garaventa A (2009). The International Neuroblastoma Risk Group (INRG) classification system: an INRG Task Force report. J Clin Oncol.

[41] Maris JM, Guo C, White PS, Hogarty MD, Thompson PM, Stram DO, Gerbing R, Matthay KK, Seeger RC, Brodeur GM (2001). Allelic deletion at chromosome bands 11q14–23 is common in neuroblastoma. Med Pediatr Oncol.

[42] Mosse Y, Greshock J, King A, Khazi D, Weber BL, Maris JM (2003). Identification and high-resolution mapping of a constitutional 11q deletion in an infant with multifocal neuroblastoma. Lancet Oncol.

[43] Selzer RR, Richmond TA, Pofahl NJ, Green RD, Eis PS, Nair P, Brothman AR, Stallings RL (2005). Analysis of chromosome breakpoints in neuroblastoma at sub-kilobase resolution using fine-tiling oligonucleotide array CGH. Genes Chromosomes Cancer.

[44] Bednarski JJ, Sleckman BP (2012). Lymphocyte development: integration of DNA damage response signaling. Adv Immunol.

[45] Michels E, Hoebeeck J, De Preter K, Schramm A, Brichard B, De Paepe A, Eggert A, Laureys G, Vandesompele J, Speleman F (2008). CADM1 is a strong neuroblastoma candidate gene that maps within a 3.72 Mb critical region of loss on 11q23. BMC Cancer.

[46] Ando K, Ohira M, Ozaki T, Nakagawa A, Akazawa K, Suenaga Y, Nakamura Y, Koda T, Kamijo T, Murakami Y, Nakagawara A (2008). Expression of TSLC1, a candidate tumor suppressor gene mapped to chromosome 11q23, is downregulated in unfavorable neuroblastoma without promoter hypermethylation. Int J Cancer.

[47] De Preter K, Vandesompele J, Menten B, Carr P, Fiegler H, Edsjö A, Carter NP, Yigit N, Waelput W, Van Roy N, Bader S, Påhlman S, Speleman F (2005). Positional and functional mapping of a neuroblastoma differentiation gene on chromosome 11. BMC Genomics.

[48] Kuramochi M, Fukuhara H, Nobukuni T, Kanbe T, Maruyama T, Ghosh HP, Pletcher M, Isomura M, Onizuka M, Kitamura T, Sekiya T, Reeves RH, Murakami Y (2001). TSLC1 is a tumor-suppressor gene in human non-small-cell lung cancer. Nat Genet.

[49] Betts DR, Cohen N, Leibundgut KE, Kühne T, Caflisch U, Greiner J, Traktenbrot L, Niggli FK (2005). Characterization of karyotypic events and evolution in neuroblastoma. Pediatr Blood Cancer.

[50] Varley JM (2003). Germline TP53 mutations and Li-Fraumeni syndrome. Hum Mutat.

[51] Soussi T, Dehouche K, Béroud C (2000). p53 website and analysis of p53 gene mutations in human cancer: forging a link between epidemiology and carcinogenesis. Hum Mutat.

[52] Nichols KE, Malkin D, Garber JE, Fraumeni JF, Li FP (2001). Germ-line p53 mutations predispose to a wide spectrum of early-onset cancers. Cancer Epidemiol Biomarkers Prev.

[53] Bown N, Cotterill S, Lastowska M, O'Neill S, Pearson AD, Plantaz D, Meddeb M, Danglot G, Brinkschmidt C, Christiansen H, Laureys G, Speleman F, Nicholson J (1999). Gain of chromosome arm 17q and adverse outcome in patients with neuroblastoma. N Engl J Med.

[54] Van Maerken T, Vandesompele J, Rihani A, De Paepe A, Speleman F (2009). Escape from p53-mediated tumor surveillance in neuroblastoma: switching off the p14(ARF)-MDM2-p53 axis. Cell Death Differ.

[55] Van Maerken T, Rihani A, Van Goethem A, De Paepe A, Speleman F, Vandesompele J (2014). Pharmacologic activation of wild-type p53 by nutlin therapy in childhood cancer. Cancer Lett.

[56] Vassilev LT, Vu BT, Graves B, Carvajal D, Podlaski F, Filipovic Z, Kong N, Kammlott U, Lukacs C, Klein C, Fotouhi N, Liu EA (2004). *In vivo* activation of the p53 pathway by small-molecule antagonists of MDM2. Science.

[57] Gumy-Pause F, Wacker P, Maillet P, Betts D, Sappino AP (2003). ATM gene alterations in childhood acute lymphoblastic leukemias. Hum Mutat.

[58] Gumy-Pause F, Ozsahin H, Khoshbeen-Boudal M, Betts DR, Maillet P, Sappino AP (2008). Detection of ATM gene deletion/duplication by multiplex ligation-dependant probe amplification in childhood lymphoid malignancies: a report from the Children's Oncology Group. Leuk Res.

[59] Gumy-Pause F, Pardo B, Khoshbeen-Boudal M, Ansari M, Gayet-Ageron A, Sappino AP, Attiyeh EF, Ozsahin H (2012). GSTP1 hypermethylation is associated with reduced protein expression, aggressive disease and prognosis in neuroblastoma. Genes Chromosomes Cancer.

[60] Sappino AP, Buser R, Lesne L, Gimelli S, Béna F, Belin D, Mandriota SJ (2012). Aluminium chloride promotes anchorage-independent growth in human mammary epithelial cells. J Appl Toxicol.

