# Supplementary material for: Ataxia-telangiectasia mutated (ATM) silencing promotes neuroblastoma progression through a MYCN independent mechanism
Source: Oncotarget. 2015 May 26;6(21):18558–76. doi: 10.18632/oncotarget.4061 (PMC4621910; doi:10.18632/oncotarget.4061)
Supplement: Supplementary file 1 [file oncotarget-06-18558-s001.pdf]

SUPPLEMENTARY FIGURES

Sample	INSS Stage	*MYCN* status	11q LOH	*ATM* rare sequence variants	*ATM* MLPA	*ATM* methylation status
1	1	Not ampl	NI	c.8419–21A>G	no deletion	UM
2	4	Not ampl	LOH		no deletion	UM
3	4	Not ampl	No LOH		no deletion	UM
4	3	Not ampl	LOH		*ATM* deletion	UM
5	4	Not ampl	No LOH		no deletion	UM
6	1	Not ampl	No LOH		no deletion	UM
7	4	Not ampl	No LOH		no deletion	UM
8	2	Not ampl	No LOH		no deletion	UM
9	4	Not ampl	LOH		*ATM* deletion	UM
10	4	Ampl	No LOH		*ATM* duplication	UM
11	3	Not ampl	No LOH		no deletion	UM
12	4	Not ampl	LOH		*ATM* deletion	UM
13	4	Not ampl	LOH		*ATM* deletion	UM
14	3	Not ampl	No LOH		no deletion	UM
15	1	Not ampl	No LOH		no deletion	UM
16	4	Ampl	No LOH		no deletion	UM
17	2	Not ampl	No LOH		no deletion	UM
18	4	Not ampl	LOH		no deletion	UM
19	4	Not ampl	No LOH		no deletion	UM
20	4	Not ampl	LOH	*c.1810C>T*[°]<br>*c.4388T>G*[*]	*ATM* deletion	UM
21	3	Ampl	No LOH		no deletion	UM
22	2	Not ampl	No LOH	c.1810C>T[*]	no deletion	UM
23	4	Not ampl	LOH		*ATM* deletion	UM
24	2	Not ampl	No LOH		no deletion	UM
25	4	Not ampl	LOH		*ATM* deletion	UM
26	3	Ampl	No LOH		no deletion	UM
27	4	Not ampl	No LOH		no deletion	UM
28	4	Not ampl	LOH		*ATM* deletion	UM
29	1	Not ampl	No LOH		no deletion	UM
30	1	Not ampl	LOH	*c.5177+62C>A*[*]	*ATM* deletion	UM
31	4	Not ampl	LOH		*ATM* deletion	UM
32	4	Not ampl	No LOH		no deletion	UM

(*Continued*)

Sample	INSS Stage	*MYCN* status	11q LOH	*ATM* rare sequence variants	*ATM* MLPA	*ATM* methylation status
33	1	Not ampl	No LOH		no deletion	UM
34	4	Not ampl	LOH		*ATM* deletion	UM
35	4	Ampl	LOH		no deletion	UM
36	4	Ampl	No LOH	UTR(-425)C>A*	no deletion	UM
37	4	Ampl	No LOH		no deletion	UM
38	4	Ampl	LOH		*ATM* deletion	UM
39	3	Not ampl	No LOH		no deletion	UM
40	4	Not ampl	LOH		*ATM* deletion	UM
41	2	Not ampl	NA		no deletion	UM
42	2	Not ampl	NA		no deletion	UM
43	1	Not ampl	NI		*ATM* deletion	UM
44	1	Not ampl	NA	c.1810C>T	no deletion	UM
45	2	Not ampl	NA		no deletion	UM
46	2	Not ampl	NA		no deletion	UM
47	1	Not ampl	NA		no deletion	UM
48	1	Not ampl	NA		no deletion	UM
49	1	Not ampl	NA		no deletion	UM
50	1	Not ampl	NA		no deletion	UM

Italic: tumoral homozygous alteration;
*germline heterozygote variant;
°germline homozygote variant;
UM: unmethylated; LOH: loss of heterozygosity; Ampl: amplified; Not ampl: not amplified; NA: not available; NI: not informative.

Supplementary Figure S1: Summary of the *ATM* alterations found in NB tumor samples.

variable 1	Hazard ratio (95% CI)	*p*-value	variable 2	Hazard ratio (95% CI)	*p*-value	
**Complete 110 patient cohort**						
**univariate**						
ATM loss (yes vs. no)	2.0 (1.0–3.8)	0.0449				
MYCN amplification (yes vs. no)	6.6 (3.4–12.9)	3.86E-08				
MYCN-157 signature (pos vs. neg)	22.5 (6.8–73.8)	2.92E-07				
Stage (1, 2, 4s vs. 3, 4)	37.0 (5.1–270.6)	0.000377				
**multivariate**						
ATM loss (yes vs. no)	4.1 (1.9–8.7)	0.000204	MYCN amplification (yes vs. no)	11.5 (5.4–24.7)	3.19E-10	independent
ATM loss (yes vs. no)	1.4 (0.7–2.6)	0.369	MYCN-157 signature (pos vs. neg)	21.5 (6.5–70.9)	4.76E-07	
ATM loss (yes vs. no)	1.5 (0.8–2.8)	0.268198	Stage (1, 2, 4s vs. 3, 4)	35.1 (4.8–257.4)	4.67E-04	
**non MYCN amplified patient cohort**						
**univariate**						
ATM loss (yes vs. no)	7.8 (2.6–23.4)	0.000253				
MYCN-157 signature (pos vs. neg)	17.1 (5.0–58.8)	6.72E-06				
Stage (1, 2, 4s vs. 3, 4)	26.1 (3.5–195.1)	0.0015				
**multivariate**						
ATM loss (yes vs. no)	4.0 (1.3–12.3)	0.017516	MYCN-157 signature (pos vs. neg)	11.4 (3.2–40.9)	0.000174	independent
ATM loss (yes vs. no)	4.9 (1.6–14.9)	0.00509	Stage (1, 2, 4s vs. 3, 4)	18.4 (2.4–139.5)	0.00492	independent

Supplementary Figure S2: Cox regression for survival analysis.

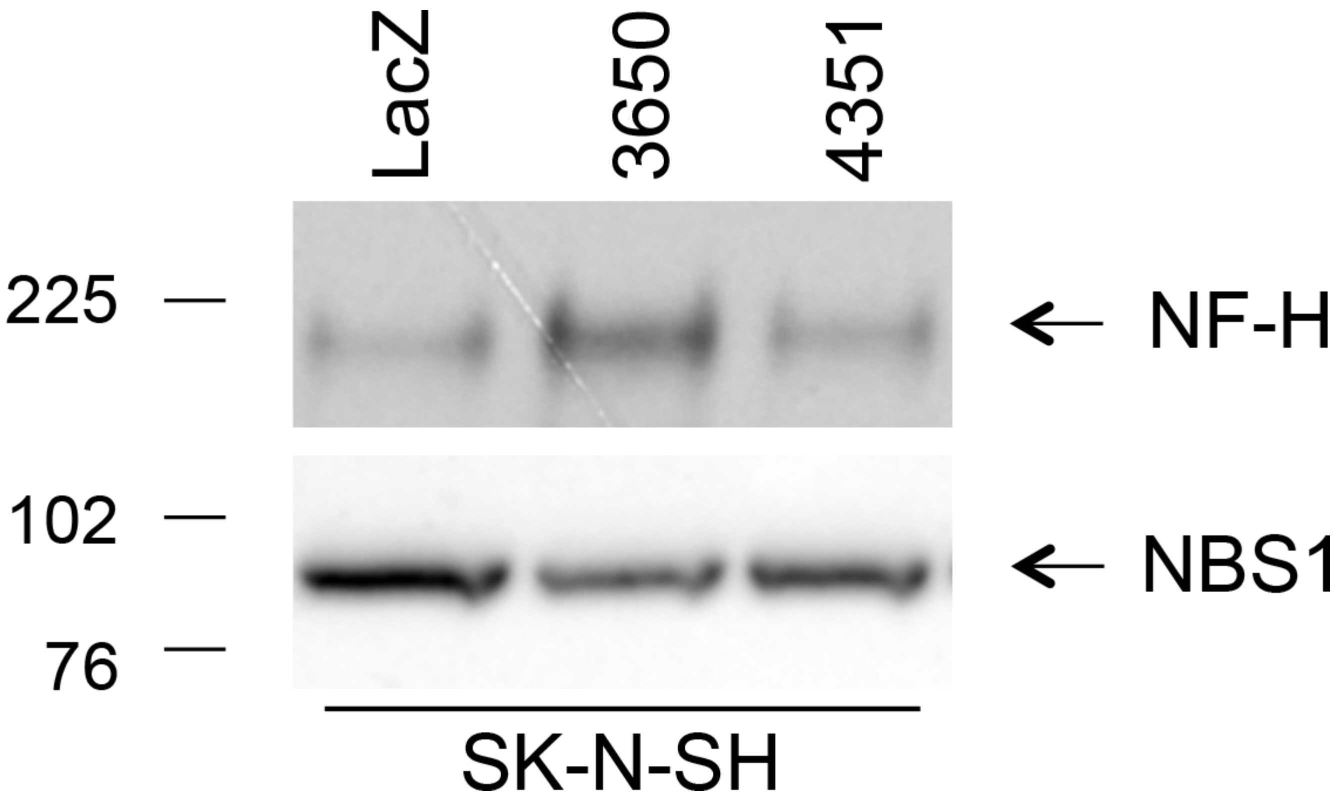

Supplementary Figure S3: Western Blotting for NF-H in SK-N-SH cells stably transfected with *ATM* shRNA vector 3650, 4351 or with a LacZ shRNA vector as a control. NBS1 was used as a loading control. One of two experiments with equivalent results is shown. Numbers on the left indicate kDa. NF-H antibody (cat. no. ab40796) was from Abcam.

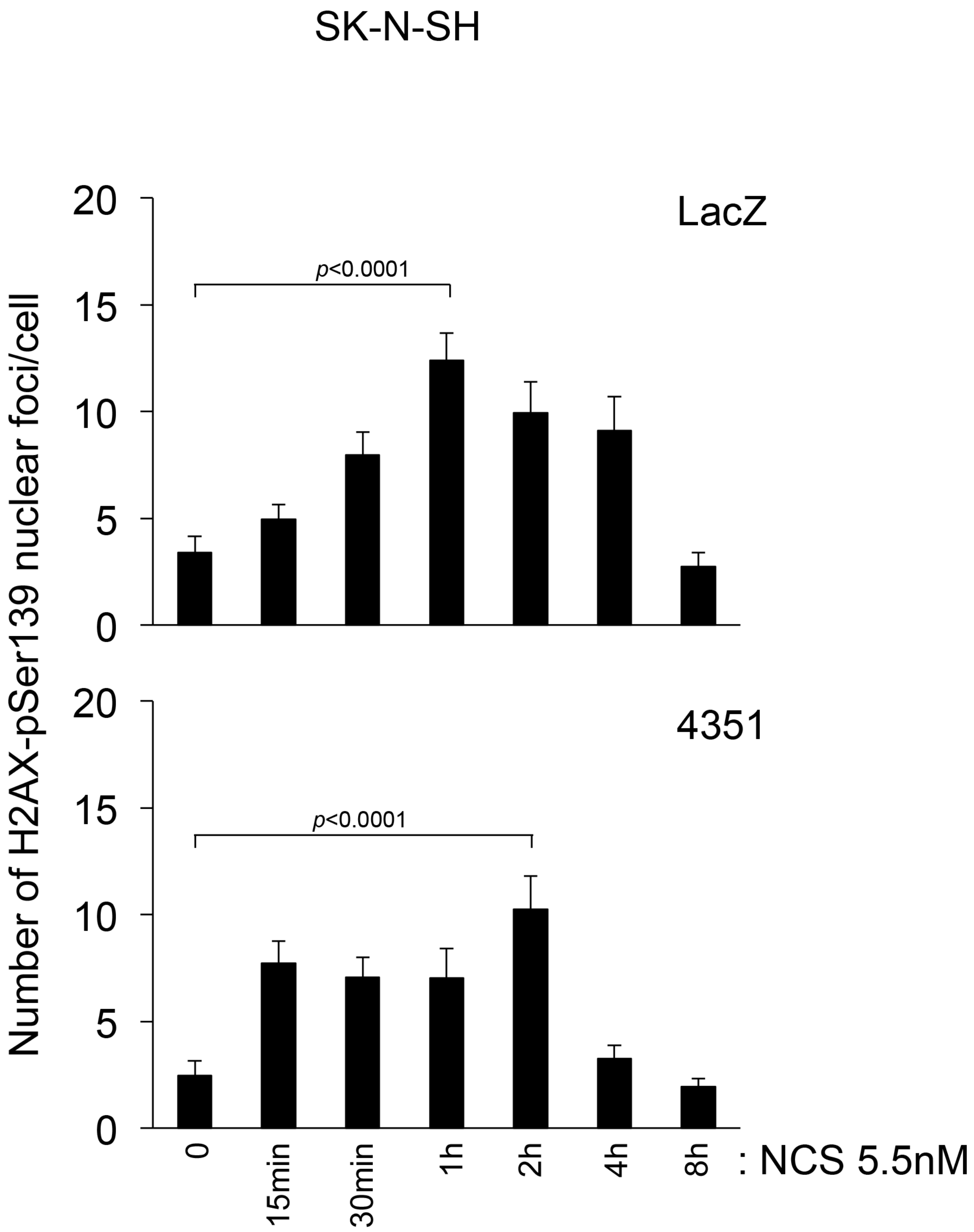

Supplementary Figure S4: Quantification of H2AX-pSer139 nuclear foci in NCS treated SK-N-SH stable transfectants. Approximately 70% confluent cultures of SK-N-SH cells stably transfected with *ATM* shRNA vector 4351 or with a LacZ shRNA vector as a control were stained for H2AX-pSer139 nuclear foci at the indicated times after treatment with NCS 5.5 nM. The graphs show the mean number of H2AX-pSer139 nuclear foci/cell ± SEM from at least 40 counted cells/condition. *p* values in the Figure refer to two-tailed *t*-test.

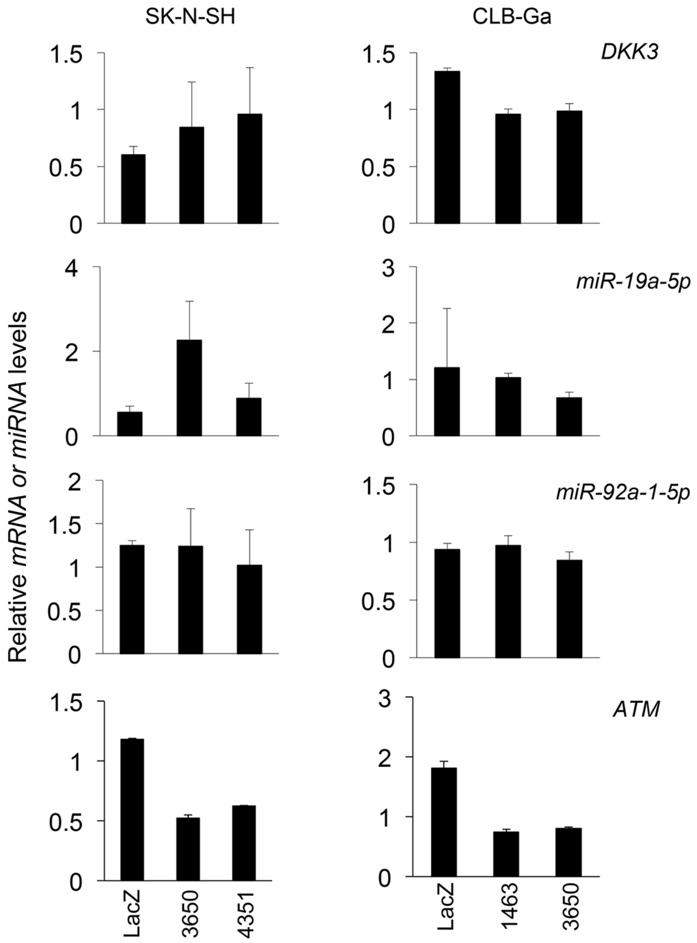

Supplementary Figure S5: The graphs show the normalized relative mRNA or miRNA levels ± SD from two different experiments for *DKK3***,** miR-19a-5p, miR-92a-1–5p or *ATM,* as assessed by real-time quantitative PCR, in SK-N-SH cells or CLB-Ga cells stably transfected with *ATM* shRNA vectors 1463, 3650, 4351 or with LacZ shRNA vector as indicated.

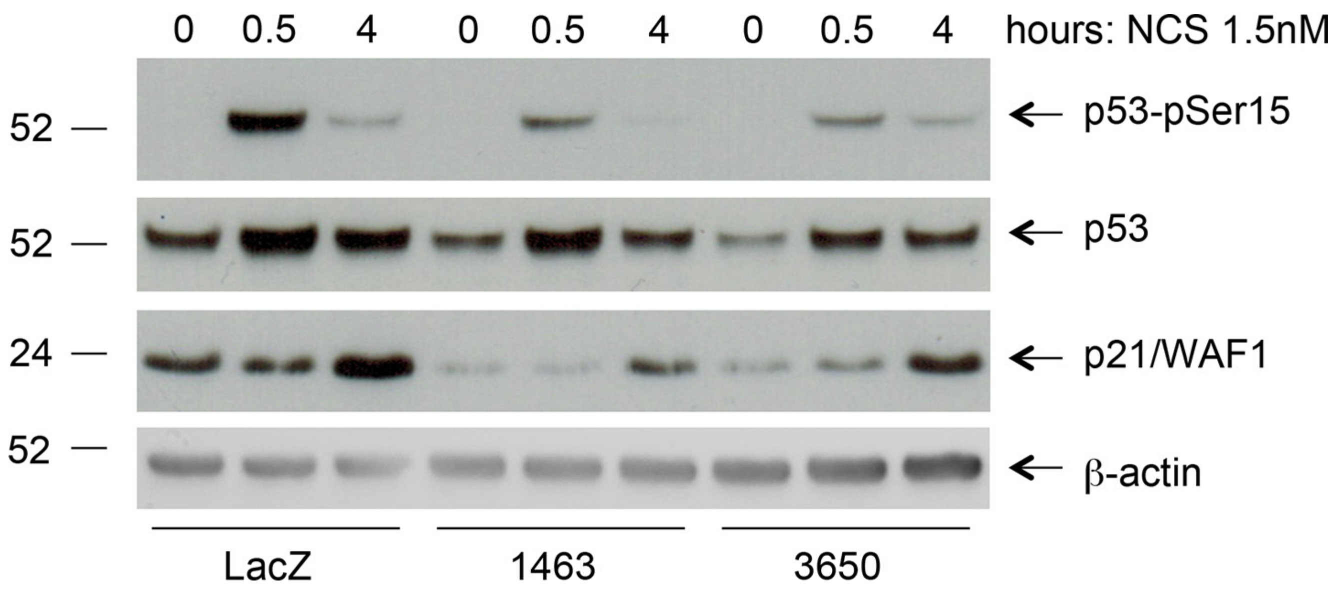

Supplementary Figure S6: GI-ME-N cells stably transfected with *ATM* shRNA vectors 1463, 3650 or with a LacZ shRNA vector as a control were incubated in the presence of NCS 1.5 nM for the indicated time points and analyzed for p53-pSer15, p53, p21/WAF1 or β-actin levels by Western Blotting.
